# Limpet‐Inspired Multifunctional Composite Fibers with Exceptional Mechanical Performance From Self‐Assembled Aramid Nanofibers

**DOI:** 10.1002/advs.202509321

**Published:** 2025-08-26

**Authors:** Tiantian Ren, Shouhua Feng, Ming Yang

**Affiliations:** ^1^ The State Key Laboratory of Inorganic Synthesis and Preparative Chemistry College of Chemistry Jilin University Changchun 130012 China

**Keywords:** aramid nanofibers, composite fibers, limpet teeth, mineralization, self‐assembly

## Abstract

Aramid nanofibers (ANFs) have emerged as promising building blocks for bioinspired materials due to their exceptional mechanical strength and chemical stability. However, their widespread application has been hindered by the limitations of conventional top‐down synthesis from Kevlar, which is costly, inefficient, and offers limited control over molecular structure. Addressing this challenge, a cost‐effective, bottom‐up strategy is reported to fabricate limpet‐inspired composite fibers by combining the self‐assembly of ANFs from poly(paraphenylene terephthalamide) polyanions with in situ iron oxide mineralization. This approach enables spontaneous co‐alignment of ANFs and β‐FeOOH nanowhiskers, emulating the hierarchical architecture of limpet teeth. Guided by hydrogen bonding and *π–π* stacking, self‐assembly regulates nanoscale ordering, crystallinity, and interfacial interactions—critical for enhancing mechanical performance. The resulting fibers exhibit an ultimate strength of 1.8 GPa, modulus of 42.7 GPa, and toughness of 336 MJ m^−^^3^, surpassing spider silk and many high‐performance synthetic fibers. Multiscale toughening mechanisms, including crystallographic slip, melt‐recrystallization, and micro‐crazing, are enabled by the semicrystalline structure of the ANFs. Beyond mechanical properties, the fibers display paramagnetic behavior and UV resistance. This work introduces a scalable platform for multifunctional composite fibers, integrating molecular‐level control with structural biomimicry and advanced functionality.

## Graphical abstract

Inspired by the architecture of limpet teeth, composite fibers formed via self‐assembled aramid nanofibers and in situ iron mineralization achieve exceptional mechanical performance and multifunctionality, surpassing many natural and synthetic benchmarks.

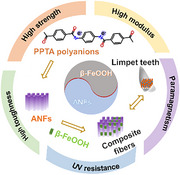

## 1 Introduction

The unique structure of teeth has inspired numerous artificial designs, enabling the development of previously unachievable sets of properties,^[^ ^1^ ^]^ thus filling the unexplored regions of the Ashby plot.^[^ ^2^, ^3^, ^4^, ^5^, ^6^, ^7^ ^]^ Limpet teeth, in particular, have garnered significant attention due to their remarkable robustness, combining high modulus, strength, and toughness.^[^ ^8^, ^9^ ^]^ Unlike human teeth, which have a complex hierarchical architecture,^[^ ^10^ ^]^ the exceptional mechanical properties of limpet teeth arise from a distinctive composite structure—densely packed chitin fibers interwoven with filamentous crystals of iron oxide, specifically in the form of goethite (α‐FeO(OH)).^[^ ^11^, ^12^ ^]^ The alignment of these goethite minerals, controlled by chitin, is crucial to the strength of limpet teeth.^[^ ^13^, ^14^ ^]^ Recent synthetic efforts to replicate the structure of limpet teeth have been made;^[^ ^15^ ^]^ however, the performance of these artificial materials remains unsatisfactory due to challenges such as the lack of co‐alignment between two anisotropic nanostructures,^[^ ^16^ ^]^ insufficient control over the interface,^[^ ^17^ ^]^ and the poor mechanical properties of the polymer matrix.^[^ ^18^ ^]^

Aramid nanofibers (ANFs), which we introduced over a decade ago,^[^ ^19^ ^]^ have emerged as a foundational building block for high‐performance nanocomposites due to their exceptional mechanical strength, thermal stability, and chemical resistance.^[^ ^20^, ^21^, ^22^, ^23^, ^24^, ^25^, ^26^, ^27^, ^28^, ^29^ ^]^ Their structural similarity to fibrous proteins has enabled their use in a variety of biomimetic systems, including cartilage‐like hydrogels,^[^ ^30^ ^]^ nacre‐inspired layered materials,^[^ ^31^ ^]^ and cellular structures.^[^ ^32^ ^]^ While ANF‐based composites have demonstrated impressive property combinations, most studies have primarily focused on their role as stiff and strong reinforcements.^[^ ^33^, ^34^ ^]^ In contrast, their capacity to contribute to energy dissipation and toughness—key features in biological materials—remains underexplored. Our recent findings suggest that mimicking natural fiber architectures, such as the crimped morphology of collagen, can endow ANF assemblies with both high toughness and thermal stability.^[^ ^35^ ^]^ However, the underlying molecular deformation mechanisms remain poorly understood. Such insights are critical for optimizing the mechanical performance of ANF‐based materials and unlocking new functionalities in bioinspired composites.

The full potential of ANFs in driving materials innovation is limited by the dominant synthesis method, which involves dissolving Kevlar and incurs high costs.^[^ ^19^, ^36^, ^37^ ^]^ While the bottom‐up co‐polymerization approach shows promise,^[^ ^38^, ^39^ ^]^ the use of additional stabilizers can compromise the intrinsic properties of ANFs. In contrast, self‐assembly—a cost‐effective method for synthesizing supramolecular nanofibers^[^ ^40^, ^41^, ^42^, ^43^ ^]^—presents a promising pathway for optimizing the use of ANFs in composite materials, particularly for their practical application in macroscale fibers.^[^ ^44^, ^45^, ^46^ ^]^ Compared with the top‐down approach, self‐assembly is more sensitive to environmental factors, thus providing additional space for controlling the structural features of ANFs. Moreover, integrating self‐assembly with mineralization processes, central to biomineralized tissue formation,^[^ ^47^, ^48^ ^]^ offers an exciting opportunity to utilize self‐assembled ANFs as the organic matrix in the development of limpet‐inspired materials.

In this study, we report the fabrication of limpet‐inspired composite fibers by inducing the self‐assembly of poly(paraphenylene terephthalamide) (PPTA) polyanions into aramid nanofibers (ANFs) during wet spinning, which simultaneously directs the oriented growth of β‐FeOOH nanowhiskers. This process yields paramagnetic fibers with co‐aligned ANFs and nanowhiskers, exhibiting enhanced resistance to UV irradiation. Their exceptional mechanical performance arises from a synergistic interplay between effective load transfer and moderated ANF crystallinity—both driven by robust interfacial interactions with the mineral phase. The resulting biomimetic architecture combines the advantages of semicrystalline, self‐assembled ANFs with aligned inorganic reinforcement, producing composite fibers with outstanding strength, stiffness, and toughness—placing them among the highest‐performing structural fibers reported to date.

## 2 Results and Discussion

### 2.1 Self‐Assembly of ANFs from PPTA Polyanions

**Figure 1:**
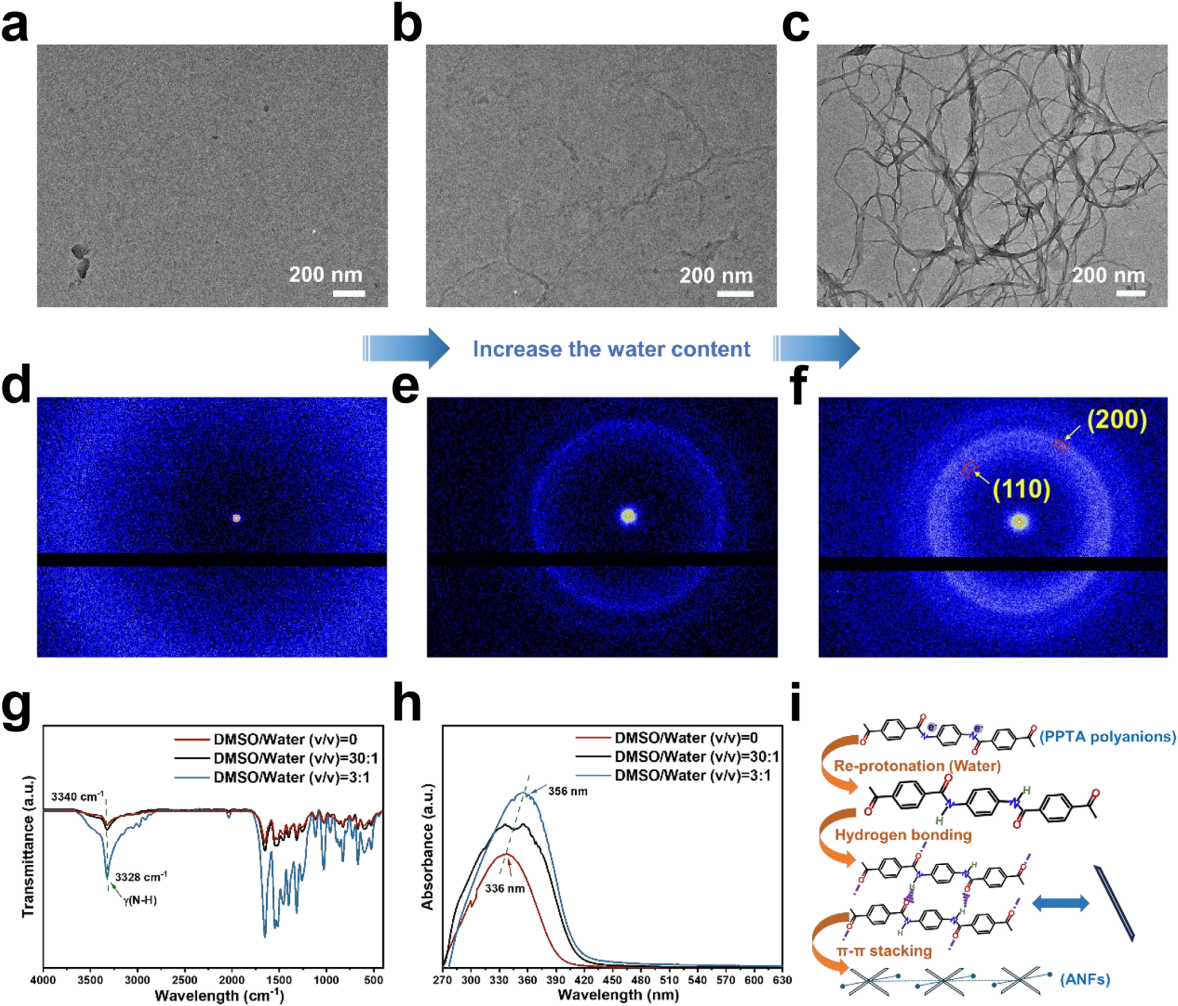
Self‐assembly of ANFs from PPTA polyanions. TEM images of PPTA polyanion DMSO solutions after the addition of varying amounts of water: a) no water, b) DMSO/water (v/v) ratio of 30:1, and c) DMSO/water (v/v) ratio of 3:1. d–f) Corresponding WAXS patterns for a–c, respectively. g) IR spectra and h) UV–vis spectra of PPTA polyanion DMSO solutions with different water additions. i) A schematic illustration of self‐assembly of ANFs from PPTA polyanions involving hydrogen bonding and aromatic stacking interactions.

For the synthesis of self‐assembled ANFs, PPTA powders, the precursor for Kevlar production, were directly dissolved in a DMSO/KOH solution. Transmission electron microscopy (TEM) images (**Figure** **1**a) showed no discernible nanofibers from the dissolution of PPTA powders, different from using Kevlar as the precursor.^[^ ^19^ ^]^ Compared to Kevlar, the dissolution of PPTA powders is more prone to produce polyanions. This difference likely stems from the lower crystallinity of PPTA powders (Figure S1, Supporting Information) compared to Kevlar‐derived precursors (e.g., pulp, short fibers, or papers).^[^ ^49^, ^50^, ^51^ ^]^ The reduced structural order weakens intermolecular hydrogen bonding, promoting solvation and polyanion formation over nanofibers. In contrast, when Kevlar is used, its higher crystallinity and aligned molecular packing help maintain fibrillar integrity during dissolution, enabling nanofiber formation under similar conditions. When water, acting as a nonsolvent, was introduced to the DMSO solution of PPTA polyanions, nanofiber formation was induced (Figure 1b). The number of nanofibers increased with the volume of water added (Figure 1c). The diameter of the nanofibers also increased from 18 ± 3 nm to 27 ± 5 nm as the volume ratio of DMSO to water (v/v) decreased from 30:1 to 3:1 (Figure S2, Supporting Information).

To confirm the in situ transition from polyanions to nanofibers, we analyzed the wide‐angle X‐ray scattering (WAXS) patterns of the PPTA polyanion solutions before and after water addition. Prior to the addition of water, no distinct diffraction peaks were observed, indicating the absence of crystalline domains and suggesting a disordered structure (Figure 1d). After adding water, diffraction rings appeared in the 2D patterns (Figure 1e), and characteristic crystal planes corresponding to ANFs, such as (200) and (110),^[^ ^52^ ^]^ gradually emerged and became more prominent as water content increased (Figure 1f). The Guinier plot based on small‐angle X‐ray scattering (SAXS) further revealed that in the low‐q region, the addition of water caused an upturn in the scattering curve, deviating from linearity (Figure S3, Supporting Information). This observation aligns with the transition from a monodisperse (polyanions) to a polydisperse (ANFs) system.^[^ ^53^ ^]^ Infrared (IR) spectra revealed that the addition of water caused a redshift in the N─H vibration,^[^ ^21^ ^]^ indicating the re‐establishment of intermolecular hydrogen bonding (Figure 1g). Simultaneously, UV–vis spectra also showed a redshift (Figure 1h), suggesting an increase in aromatic stacking interactions.^[^ ^54^ ^]^

The formation of nanofibers is likely attributed to a reduction in repulsion between molecular chains, resulting from the decrease in negative charges upon re‐protonation (Figure 1i). This effect dominates over competing factors, such as the increased solvent polarity and decreased ionic concentration induced by water addition. The re‐protonation promotes hydrogen bonding between the N─H and C═O groups, leading to the formation of larger hydrogen‐bonded sheets that enhance the aromatic stacking interactions due to the better planarity.^[^ ^35^ ^]^ These two primary intermolecular interactions cooperatively induce the formation of nanofibers (Figure 1i). Many peptide‐derived nanofibers formed through self‐assembly also rely on hydrogen bonding and aromatic interactions.^[^ ^54^, ^55^, ^56^, ^57^ ^]^ However, unlike the mechanism of supramolecular polymerization, where aromatic stacking typically drives the elongation of nanofibers,^[^ ^58^, ^59^ ^]^ the length of ANFs is pre‐determined by the molecular weight of the PPTA polyanions, with aromatic stacking primarily contributing to the increase in their thickness. The diameter of ANFs is self‐limited by the balance between electrostatic repulsion^[^ ^60^, ^61^ ^]^ and attractive forces.^[^ ^62^ ^]^

X‐ray diffraction (XRD) patterns of ANFs obtained by precipitation with excess water indicated low crystallinity (Figure S4, Supporting Information). The local ordering of the ANFs may be disrupted at the nanoscale due to limited space for crystallization.^[^ ^63^, ^64^, ^65^ ^]^ While the addition of other protic solvents, such as methanol, also facilitates nanofiber formation (Figure S5, Supporting Information), crystallinity was further reduced (Figure S4, Supporting Information). This is because methanol, as a nonsolvent, has a weaker ability to provide hydrogen for re‐protonation compared to water. The sensitivity of ANF crystallinity to nonsolvent choice aligns with the molecular assembly process involved in ANF formation.

### 2.2 Limpet Teeth‐Like Composite Fibers

**Figure 2:**
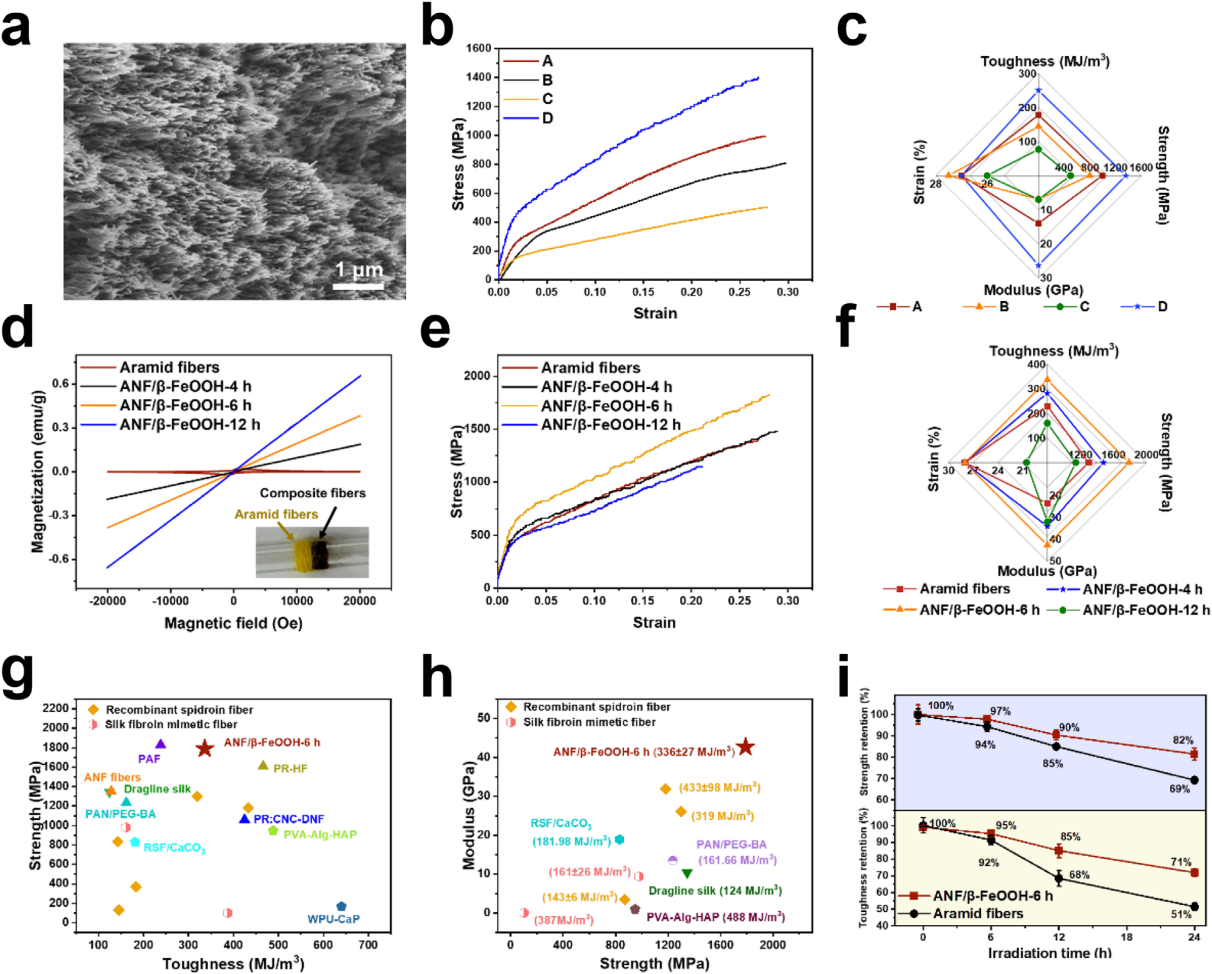
Physicochemical properties of aramid fibers and composite fibers from wet spinning. a) A cross‐sectional SEM image of aramid fibers after uniaxial constrained drying at room temperature. b) Stress–strain curves and c) summary of tensile properties of aramid fibers obtained under different conditions (the dope concentration and the spinning speed are 5 mg mL^−1^ and 10 mL h^−1^, 8 mg mL^−1^ and 10 mL h^−1^, and 10 mg mL^−1^ and 10 mL h^−1^, for A, B, and C, respectively. The spinning condition for D is the same as A, except that a post‐stretching process was applied with a stretching ratio of 10%. d) Magnetic hysteresis curves of aramid fibers and composite fibers. The inset is an optical image of aramid fibers and ANF/β‐FeOOH‐6 h. e) Stress–strain curves and f) summary of tensile properties of aramid fibers and composite fibers. Ashby plot of g) strength versus toughness and h) modulus versus strength for different materials (the details for comparison can be found in Table S3, Supporting Information). i) The change of strength and toughness of aramid fibers and ANF/β‐FeOOH‐6h after UV light irradiation for different lengths of time.

The self‐assembly of ANFs from PPTA polyanions provides an economical and effective pathway for synthesizing polymer fibers via wet spinning.^[^ ^35^, ^45^, ^46^ ^]^ In a typical synthesis, DMSO solutions of PPTA polyanions were injected through a syringe into a coagulation solution of water. During this process, re‐protonation of the polyanions induced the formation of hydrogel fibers, which could be post‐stretched using a roller. After a complete solvent exchange through immersion in water, uniaxial constrained drying was applied to remove the remaining water and obtain aramid fibers. The drying process could affect the crystallinity of ANFs due to the removal of water from the defect planes.^[^ ^35^ ^]^ The porous fibrous structures of the hydrogel fibers, revealed after freeze‐drying, were maintained even after post‐stretching (Figure S6, Supporting Information). This is reasonable given that the ANFs formed in the water bath through self‐assembly, in contrast to those directly used in conventional wet spinning methods.^[^ ^44^ ^]^ Interestingly, when dried at room temperature with fixed ends, the resulting fibers exhibited axial alignment of the ANFs, which could cluster into bundles due to the capillary forces (**Figure** **2**a). It is likely that during the uniaxial constrained drying process, the network of ANFs undergoes affine nanoscale deformation^[^ ^66^ ^]^ along the radial direction, leading to the formation of an anisotropic structure. Because the fiber length is maintained, an axial force is generated during drying, counteracting the contraction of the nanofibers along the fiber axis,^[^ ^35^ ^]^ which in turn promotes their alignment. The significant reduction in fiber diameter densifies the fiber structure, which is favored by interfibrillar interactions, further reducing voids.

Aramid fibers synthesized with a dope concentration of 5 mg mL^−1^ and a spinning speed of 10 mL h^−1^ demonstrated the most promising tensile properties, exhibiting an ultimate strength of 0.9 ± 0.1 GPa, a modulus of 14.1 ± 0.9 GPa, and toughness of 178 ± 12 MJ m^−^^3^ (Figure 2b,c). Increasing the dope concentration (Figure 2b,c) negatively impacted the mechanical properties, likely due to an increase in fiber diameter (Table S1, Supporting Information), which reduced the alignment of the ANFs, as characterized by Herman's orientation factor (Figure S7 and Table S2, Supporting Information). The effect of spinning speed on mechanical properties did not show a clear trend (Table S1, Supporting Information), likely because it influences both the fiber diameter and the shear force during spinning. Post‐stretching at a 10% stretching ratio significantly improved the fibers' properties thanks to the improved alignment (Figure S7 and Table S2, Supporting Information), resulting in an ultimate strength of 1.3 ± 0.1 GPa, a modulus of 26.3 ± 0.5 GPa, and toughness of 239 ± 11 MJ m^−^^3^ (Figure 2b,c). However, a larger stretching ratio resulted in a decrease (Table S1, Supporting Information), likely due to structural damage to the hydrogel fibers. Aramid fibers obtained using Kevlar as the precursor showed marginally superior mechanical properties (Figure S8, Supporting Information), attributable to the enhanced alignment of pre‐formed ANFs during shear‐induced assembly.

To synthesize limpet teeth‐like composite fibers, the optimal conditions for wet spinning aramid fibers were used, except that the coagulation bath was replaced with a Fe^3^⁺ solution (0.005 mol L^−1^). Like aramid fibers, composite fibers could be produced continuously using a home‐built spinning device (Figure S9, Supporting Information). Since PPTA polyanion solutions are highly basic, β‐FeOOH formed simultaneously along with the self‐assembly of ANFs. Composite fibers, immersed in the Fe^3+^ solution for varying durations, were labeled as ANF/β‐FeOOH‐*x* h, where “*x*” (4, 6, or 12) denotes the immersion time in hours. The intensity of X‐ray diffraction peaks from β‐FeOOH increased with longer immersion times (Figure S10, Supporting Information), implying higher amounts of β‐FeOOH.

The magnetic properties shifted from diamagnetic for aramid fibers to paramagnetic for composite fibers (Figure 2d) due to the involvement of β‐FeOOH nanowhiskers,^[^ ^67^ ^]^ and the color changed from yellow to dark brown (Figure 2d, inset). The magnetic susceptibility of composite fibers increased with increasing *x*, as evidenced by the change of slope in the magnetic hysteresis curves (Figure 2d). While the stress–strain curve of ANF/β‐FeOOH‐4 h roughly overlapped with aramid fibers (Figure 2e,f), ANF/β‐FeOOH‐6 h exhibited an ultimate strength of 1.8 ± 0.1 GPa, toughness of 336 ± 27 MJ m^−^^3^, and a modulus of 42.7 ± 0.5 GPa (Figure 2e,f), representing increases of 38%, 41%, and 62%, respectively, compared to aramid fibers. However, extending the immersion time to 12 h led to a noticeable reduction in both strength and toughness of ANF/β‐FeOOH‐12 h (Figure 2e,f).

The combination of ultrahigh strength and toughness makes ANF/β‐FeOOH‐6 h highly competitive, even when compared to spider silk,^[^ ^68^, ^69^ ^]^ and to recently developed strong and tough polymer fibers, including polyacrylonitrile (PAN) fibers,^[^ ^70^ ^]^ polyrotaxane (PR) fibers,^[^ ^71^, ^72^ ^]^ polyacrylic acid (PA) fibers^[^ ^73^ ^]^ and composite fibers made from calcium minerals^[^ ^74^, ^75^, ^76^ ^]^ (Figure 2g; Table S3, Supporting Information). Notably, the strength and toughness of ANF/β‐FeOOH‐6 h are both significantly higher than those of aramid fibers spun directly from ANFs derived from Kevlar^[^ ^44^ ^]^ (Figure 2g; Table S3, Supporting Information), not to mention the added cost‐effectiveness. Although the strength of ANF/β‐FeOOH‐6 h is lower than that of heterocyclic aramid fibers reinforced with single‐walled carbon nanotubes,^[^ ^77^ ^]^ its toughness is much higher. ANF/β‐FeOOH‐6 h also exhibits both higher strength and toughness than most polymer fibers derived from recombinant spidroins,^[^ ^78^, ^79^, ^80^, ^81^, ^82^ ^]^ with the exception of one made from “supramolecular polyamide”, which demonstrates superior toughness but significantly lower strength^[^ ^82^ ^]^ (Figure 2g; Table S3, Supporting Information). The exceptional combination of strength and toughness—surpassing that of certain fibers derived from silk fibroin mimetics^[^ ^83^, ^84^ ^]^ (Figure 2g; Table S3, Supporting Information)—is complemented by a remarkably high modulus, which is an order of magnitude greater than that of spider silk.^[^ ^68^ ^]^ Intriguingly, the Ashby plot of strength versus modulus illustrates that ANF/β‐FeOOH‐6 h is significantly stronger and stiffer than most of the other fibers^[^ ^68^, ^70^, ^75^, ^76^, ^80^, ^81^, ^82^, ^83^, ^84^ ^]^ with a comparable level of toughness (Figure 2h; Table S3, Supporting Information). The strength (1.8 ± 0.1 GPa) and modulus (42.7 ± 0.5 GPa) of ANF/β‐FeOOH‐6 h also represent an order‐of‐magnitude improvement over bioinspired limpet tooth‐mimetic composites based on mineral‐polysaccharide colloidal liquid crystals (103.67 ± 5.94 MPa and 2.08 ± 0.46 GPa, respectively).^[^ ^17^ ^]^ The high modulus of ANF/β‐FeOOH‐6 h contributes to the superior creep resistance, consistent with the much smaller creep strain under various loading conditions than aramid fibers (Figure S11, Supporting Information).

The inclusion of β‐FeOOH nanowhiskers significantly improves resistance to UV radiation, as they efficiently absorb UV rays (Figure S12, Supporting Information), protecting the ANFs from radiation‐induced damage.^[^ ^85^ ^]^ Notably, the tensile strength and toughness of ANF/β‐FeOOH‐6 h showed minimal change after 6 h of UV exposure (Figure 2i; Figure S13 and Table S4, Supporting Information). In contrast, aramid fibers experienced a 6% reduction in tensile strength and an 8% reduction in toughness (Figure 2i; Figure S13 and Table S4, Supporting Information). After 24 h of UV exposure, aramid fibers saw a 31% decrease in tensile strength and a 49% decrease in toughness, while ANF/β‐FeOOH‐6 h exhibited much smaller decreases of 18% and 29%, respectively (Figure 2i; Figure S13 and Table S4, Supporting Information).

### 2.3 Structural Features of Composite Fibers

**Figure 3:**
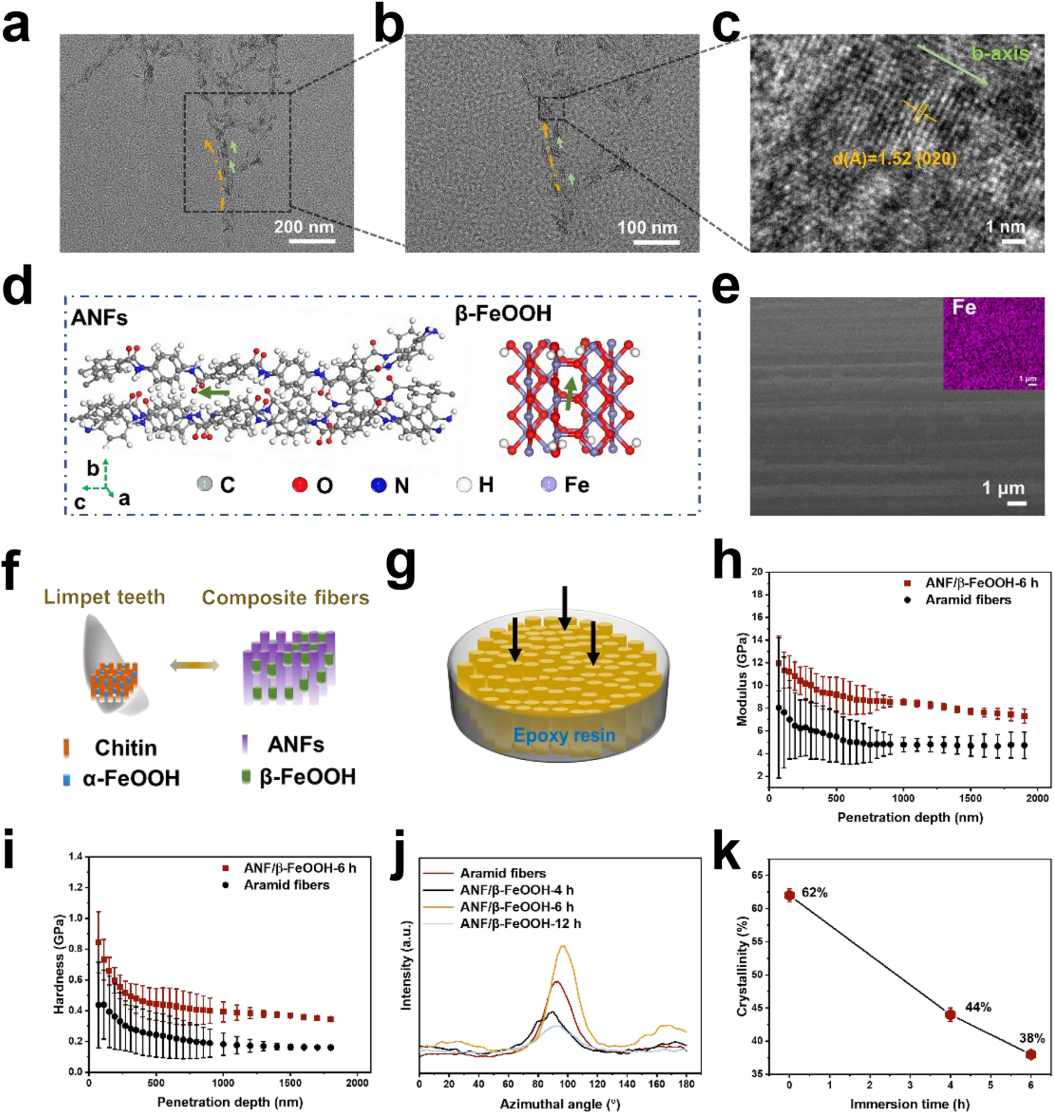
Structural characterization of composite fibers. a,b) TEM images of hydrogel fibers after 6 h immersion. The yellow and green arrows point to the axial direction of ANFs and β‐FeOOH nanowhiskers, respectively. c) An HRTEM image of hydrogel fibers after 6 h immersion. d) Dipole moments calculated based on the crystal structures of PPTA and β‐FeOOH, which are elongated along the axial direction. e) A surface SEM image of ANF/β‐FeOOH‐6h (the inset is the EDX mapping). f) Schematic illustrations of limpet teeth and composite fibers. g) A schematic of the nanoindentation test. The arrows indicate the direction of penetration. The dependence of h) modulus and i) hardness on the penetration depth. j) Azimuthal intensity profiles of (200) crystal planes from different fibers. k) The crystallinity of different fibers as a function of immersion time in the Fe^3+^ solution. 0 h corresponds to aramid fibers, while the absence of 12 h is due to the extremely weak diffraction peak from ANFs.

ANF/β‐FeOOH‐6 h contained β‐FeOOH nanowhiskers with diameters of ≈9 nm and lengths of ≈31 nm (Figure S14, Supporting Information). These nanowhiskers aligned parallel to the long axis of the ANFs, as confirmed by TEM imaging of hydrogel fibers dispersed in water (**Figure** **3**a,b). High‐resolution TEM (HRTEM) images revealed lattice fringes of 1.52 Å, corresponding to the (020) crystal planes of β‐FeOOH, consistent with growth along the b‐axis (Figure 3c). The nucleation of β‐FeOOH on the surface of ANFs is facilitated by the structural compatibility between PPTA and β‐FeOOH, as shown in our previous work using Kevlar‐derived ANFs.^[^ ^86^ ^]^ The self‐assembly of PPTA polyanions into ANFs precedes β‐FeOOH deposition during the wet spinning process, allowing the ANFs to serve as an organic matrix that promotes heterogeneous nucleation and oriented growth of β‐FeOOH.^[^ ^87^ ^]^ Our calculations further revealed that the dipole moments of both ANFs and β‐FeOOH nanowhiskers align along their long axes (Figure 3d), which favors the mutual alignment in the composite fibers. Such a configuration strengthens the dipolar interactions, guiding the system toward its lowest energy state.^[^ ^88^ ^]^ In contrast, for ANF/β‐FeOOH‐4 h, only small nanoparticles formed (Figure S15a,c, Supporting Information), while for ANF/β‐FeOOH‐12 h, the β‐FeOOH nanowhiskers continued to grow, becoming thicker and much less oriented (Figure S15b,d,e, Supporting Information).

Surface SEM images and EDX mappings revealed the uniform distribution of β‐FeOOH nanowhiskers within ANF/β‐FeOOH‐6 h (Figure 3e). In combination with cross‐sectional SEM images and TEM observations (Figures 2a and 3a,b), these results indicate that ANF/β‐FeOOH‐6 h contains β‐FeOOH nanowhiskers uniformly embedded in the interfibrous space in a coaligned arrangement, reminiscent of the structure of limpet teeth (Figure 3f). To assess the mechanical properties using nanoindentation testing to further confirm the homogeneous distribution of β‐FeOOH nanowhiskers, the fibers were encapsulated in epoxy resin, then subjected to abrasion to expose the cross‐section (Figure 3g). A statistical increase in modulus (from 4.77 ± 0.64 GPa to 8.56 ± 0.43 GPa) and hardness (from 0.18 ± 0.07 GPa to 0.39 ± 0.06 GPa) was observed when comparing the aramid fibers with ANF/β‐FeOOH‐6 h (Figure 3h,i). The lower modulus and hardness relative to limpet^[^ ^8^ ^]^ and chiton teeth^[^ ^89^ ^]^ are likely due to the limited amounts of β‐FeOOH. TGA results (Figure S16, Supporting Information) showed that the β‐FeOOH content in ANF/β‐FeOOH‐4, 6, and 12 h is ≈9.7%, 18.2%, and 27.3%, respectively.

For ANF/β‐FeOOH‐4 h, the diffraction pattern in the 2D SAXS image appeared more dispersed in the equatorial direction than the aramid fibers, while the pattern for ANF/β‐FeOOH‐6 h became more elongated (Figure S17, Supporting Information). When the immersion time was extended to 12 h, the 2D SAXS pattern became more uniformly distributed again. Consistent with these changes, the azimuthal strength of the composite fibers first decreased, then increased, and decreased once more with increasing immersion time (Figure 3j), implying the variation of alignment. The decreased alignment in ANF/β‐FeOOH‐4 h is likely due to unreacted Fe^3^⁺, which disrupts interfibrillar interactions during drying. In ANF/β‐FeOOH‐6 h, the thin nature of β‐FeOOH nanowhiskers allows them to conform to the surface of the ANFs, facilitating uniform embedment in the interfibrous space, which helps maintain better axial alignment. However, for ANF/β‐FeOOH‐12 h, the larger size of β‐FeOOH nanowhiskers and its less defined orientation led to a decrease in alignment. Increasing the concentration of Fe^3^⁺ also reduced the mechanical properties (Table S5, Supporting Information), as the nanowhiskers grew larger and became more disoriented (Figure S18, Supporting Information).

When the coagulation bath was water, the transverse crystallinity of aramid fibers was calculated to be ≈62% (Table S2 and Figure S19, Supporting Information). However, when the coagulation bath was changed to Fe^3^⁺ solutions, the characteristic diffraction peaks of ANFs weakened significantly, and the calculated crystallinity decreased to 44% for ANF/β‐FeOOH‐4 h and 38% for ANF/β‐FeOOH‐6 h (Figures 3k and S10, Supporting Information). After 12 h of immersion in the Fe^3^⁺ solution, the diffraction peaks of ANFs were barely visible (Figure S10, Supporting Information). The continuous reduction in crystallinity with increasing immersion time can be attributed to the strong interfacial interactions between ANFs and β‐FeOOH nanowhiskers. In addition to hydrogen bonding—evidenced by the redshift in the IR peak associated with N─H vibrations of ANFs following the attachment of β‐FeOOH nanowhiskers (Figure S20, Supporting Information)—XPS spectra further confirmed the formation of interfacial Fe─O─C bonds at 530.2 eV (Figure S21, Supporting Information).^[^ ^86^ ^]^ The electronegativity difference between Fe and O atoms reduces electron density around oxygen, likely inhibiting the formation of intermolecular hydrogen bonding and consequently hindering crystallization in the amorphous regions of ANFs during drying. Similar effect was observed for Fe^3+^ during the self‐assembly of ANFs (Figure S22, Supporting Information).

### 2.4 Deformation Mechanism

**Figure 4:**
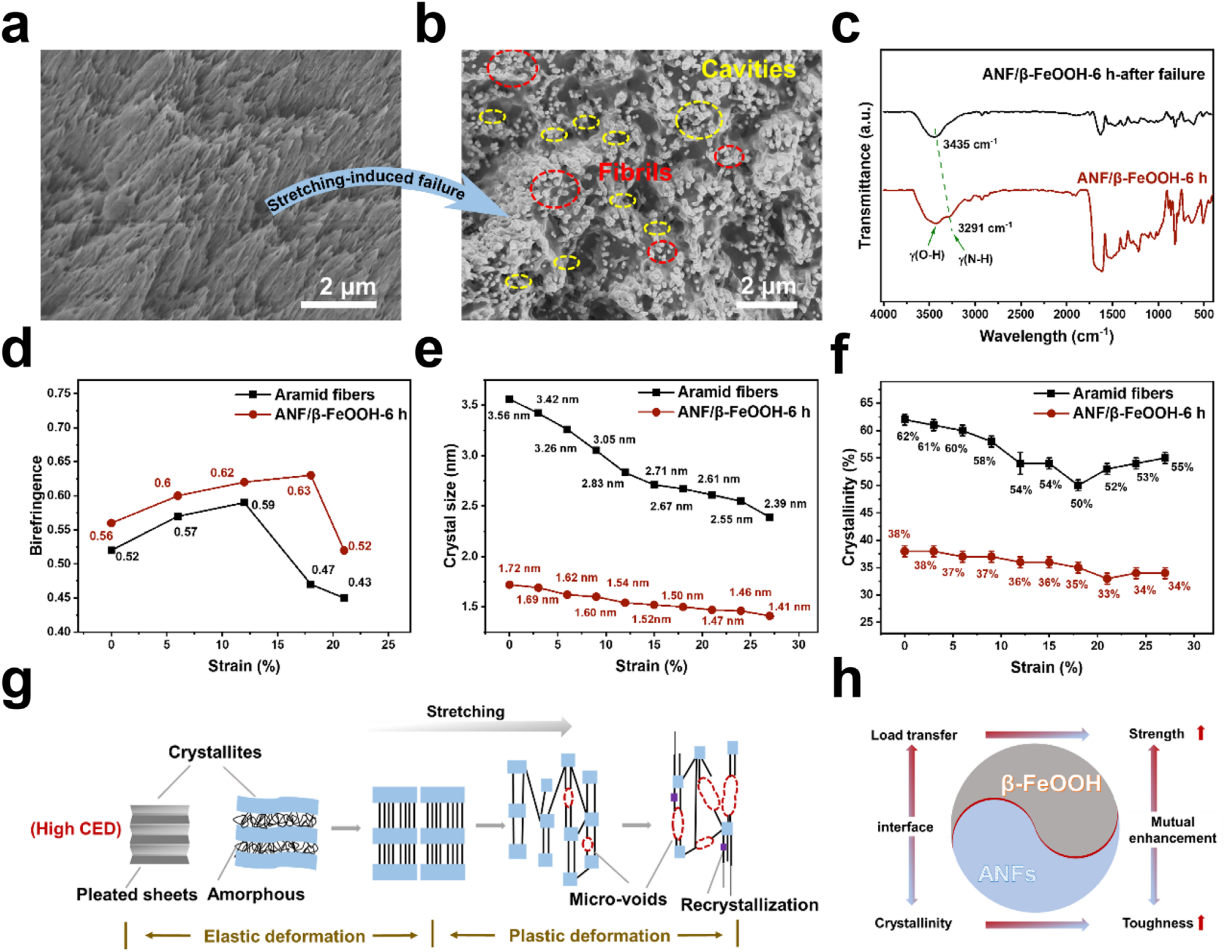
Deformation mechanism. Cross‐sectional SEM images of ANF/β‐FeOOH‐6 h a) before and b) after stretching‐induced failure. c) IR spectra of ANF/β‐FeOOH‐6 h before and after stretching‐induced failure. Evolution of d) birefringence, e) crystal size, and f) crystallinity under different strains for aramid fibers and ANF/β‐FeOOH‐6 h. g) Schematic illustration of the deformation mechanism of semicrystalline ANFs consisting of pleated sheet crystallites with high cohesive energy density (CED). h) A schematic illustrates how interfacial interactions balance load transfer and ANF crystallinity, enabling the simultaneous enhancement of strength and toughness.

A dramatic morphological change was observed in both aramid fibers and ANF/β‐FeOOH‐6 h after stretching‐induced failure (**Figure** **4**a,b; Figure S23, Supporting Information). Cavities spread across the whole section, and the fibrillar structures were found to emanate from the plastically deformed polymer matrix (Figure 4b; Figure S23, Supporting Information). These tiny fibrils are different from the original ANFs (Figure 4a) in both size and shape and are likely generated during structure failure. Aramid fibers that underwent stretching‐induced failure exhibited a noticeable blueshift in the IR peak from 3326 to 3430 cm⁻¹, corresponding to the N─H vibrational mode (Figure S24, Supporting Information).^[^ ^19^ ^]^ This shift is likely due to the breakage of hydrogen bonds,^[^ ^35^, ^90^ ^]^ resulting in the transition from an ordered to disordered structure. In ANF/β‐FeOOH‐6 h, a similar blueshift from 3291 to 3435 cm⁻¹ was observed (Figure 4c).

Under polarized optical microscopy (POM), for both aramid fibers and ANF/β‐FeOOH‐6 h, the birefringence calculated by using the Sénarmont compensation method^[^ ^35^, ^91^ ^]^ increased first due to the stretching‐induced alignment (Figure 4d). There was a significant drop in the birefringence when the strain reached ≈18% for aramid fibers and ≈21% for ANF/β‐FeOOH‐6 h, which coincides with the emergence of stress whitening (Figure S25, Supporting Information). Stress whitening is related to the cavities observed in the fractured surface (Figure 4b; Figure S23, Supporting Information), which change the refractive index and scatter lights,^[^ ^92^ ^]^ consistent with the semicrystalline nature of self‐assembled ANFs.

Real‐time 2D wide angle X‐ray diffraction (WAXD) patterns showed that tensile stretching is accompanied by a continuous reduction in the size of PPTA crystallites in both aramid fibers and ANF/β‐FeOOH‐6 h (Table S6 and Figures S26 and S27, Supporting Information). Specifically, the crystal size decreased from 3.56 to 2.39 nm for aramid fibers, and from 1.52 to 1.41 nm for ANF/β‐FeOOH‐6 h, when the strain increased from 0% to 27% (Figure 4e). In addition, the crystallinity of aramid fibers decreased from 62% to 50% when the strain reached 18%, then began to increase to 52% at a strain of 21%, and finally to 55% at a strain of 27% (Figure 4f), based on 1D data calculations (Figure S28, Supporting Information). For ANF/β‐FeOOH‐6 h, crystallinity decreased from 38% to 33% up to a strain of 21%, then slightly increased to 34% upon further stretching (Figure 4f; Figure S29, Supporting Information). The reduction of crystal size and the increase of crystallinity imply that crystallographic slip and the melt‐recrystallization process^[^ ^93^ ^]^ both occur during the stretching of ANFs, and the latter becomes important at a later stage when there is a large amount of newly formed amorphous phase.

It is hypothesized that self‐assembled ANFs with low crystallinity consist of pleated sheet crystallites^[^ ^35^ ^]^ interconnected by amorphous interlamellar layers, a typical model for semicrystalline polymers (Figure 4g). In the stress–strain curves, the initial elastic region corresponds to the extension of entangled chains and tie molecules within the amorphous phase (Figure 4g). After the yield point, the crystallographic slip mechanism becomes dominant for supplying the substantial strain energy (Figure 4g), which may also attenuate peak stress concentrations near crack tips through a deflection mechanism.^[^ ^94^ ^]^ Crystallographic slip leads to irreversible lamellar unfolding and fragmentation into smaller segments, consistent with the breakage of hydrogen bonding (Figure 4c; Figure S24, Supporting Information) and the reduction in the crystal size and crystallinity (Figure 4e,f). The nucleation of submicroscopic voids may occur due to the breaking of the amorphous phase and crystallites (Figure 4g), as observed in the fractured surface (Figure 4b; Figure S23, Supporting Information), inducing stress whitening (Figure S25, Supporting Information). Cavitation may lead to shear yielding and effectively delocalize the cracks, avoiding stress concentration.^[^ ^95^ ^]^ As the applied load increases, local strain is transferred to the crazed zone, where the newly formed fibrils are stretched.^[^ ^96^ ^]^ The craze grows through further fibril extension and continued fibrillation, eventually propagating into a crack via scission or disentanglement, leading to fiber failure (Figure 4g) and exposing the fibrous surface (Figure 4b; Figure S23, Supporting Information). The failure process may also be accompanied by the recrystallization of the amorphous phase within the drawn fibers (Figure 4g),^[^ ^93^, ^97^ ^]^ which results in increased crystallinity at the final stages of deformation (Figure 4f) and possibly double yielding.^[^ ^93^ ^]^ While the shear displacement of individual fibers may also contribute to the plastic deformation of the fibrous structure,^[^ ^98^ ^]^ it is unlikely to cause a dramatic change at the fractured surface. The lack of interfibrillar tie molecules^[^ ^99^ ^]^ and polymer matrix^[^ ^100^ ^]^ also makes this mechanism less important in the current system.

In mineralized collagen, the mineral crystals bear most of the stress, while the collagen primarily governs the material's deformation response.^[^ ^101^ ^]^ This combination allows bones to achieve exceptional energy dissipation and fracture resistance. In our case, the semicrystalline nature of self‐assembled ANFs enables multiscale toughening mechanisms—such as crystallographic slip, melt‐recrystallization, and crazing—to effectively occur during deformation, while the aligned interfibrillar β‐FeOOH nanowhiskers enhance strength by effective load transfer. There exists an optimal balance between load transfer at the interface and the altered crystallinity of self‐assembled ANFs mediated by interfacial interactions (Figure 4h), responsible for the simultaneous improvement of strength and toughness. For ANF/β‐FeOOH‐4 h, the small number of β‐FeOOH nanoparticles helps offset the decrease in alignment and crystallinity of the ANFs, causing only a minimal impact on the mechanical properties. Extending the immersion time to 6 h promotes the alignment of ANFs and β‐FeOOH nanowhiskers, enhancing load transfer and increasing both strength and modulus. Meanwhile, the reduced crystallinity of the ANFs allows for greater strain capacity for dissipating energy. After 12 h of immersion, however, the mechanical properties deteriorate due to a significant loss of alignment.

Unlike traditional semicrystalline polymers such as nylon and meta‐aramids like Nomex, the linear, rigid backbone of PPTA imparts a significantly higher cohesive energy density (CED) to its crystallites (Figure 4g; Figure S30, Supporting Information). While β‐sheet nanocrystals are known to govern the mechanical properties of spider silk,^[^ ^102^, ^103^ ^]^ the high CED in PPTA similarly explains the enhanced yield strength of ANF‐based fibers. This results from extensive hydrogen bonding and aromatic stacking interactions, which also account for their significantly higher modulus compared to spider silk. The amorphous phase further enhances mechanical integrity by bridging crystallites in a soft–hard sequence. However, unlike spider silk—whose strain‐hardening behavior stems from flexible polypeptide chains and intramolecular β‐sheets in the amorphous phase^[^ ^104^ ^]^—aramid‐based fibers lack these structural motifs. Instead, their amorphous regions facilitate robust organic–inorganic interactions via accessible functional groups, creating a reinforced interface. Critically, this interface enables precise modulation of ANF self‐assembly, allowing tailored optimization of structural and functional properties in ANF‐based materials.

## 3 Conclusion

In conclusion, limpet teeth‐inspired paramagnetic composite fibers with enhanced resistance to UV radiation are synthesized through a scalable wet spinning process that integrates the self‐assembly of ANFs with the in situ mineralization of iron oxide. The remarkable mechanical properties arise from the coalignment of ANFs and β‐FeOOH nanowhiskers, which ensures efficient load transfer through a robust interface. The semicrystalline nature of the self‐assembled ANFs, modulated by the formation of β‐FeOOH, is crucial for overcoming the trade‐off between strength and toughness. Future work could focus on optimizing the self‐assembly process of ANFs and diversifying the functions through the incorporation of other forms of iron oxide minerals.

## 4 Experimental Section

### Materials

PPTA powders were purchased from Special Textile Raw Materials Co., Ltd. (China), while dimethyl sulfoxide (DMSO), potassium hydroxide (KOH), and ferric chloride hexahydrate (FeCl₃·6H₂O) were sourced from Tianjin Tiantai Co., Ltd. and Shanghai Aladdin Bio‐Chem Technology Co., Ltd. (China). Ultrapure water (18.2 MΩ) used in all experiments was obtained from an RSJ Water Purification System (China).

### Preparation of PPTA Polyanion Solutions

1.6 g of PPTA powder and 2.4 g of KOH were added to 80 mL of DMSO and stirred for one week to form a dark red PPTA polyanion solution (20 mg mL^−1^). Solutions with different concentrations were then prepared by dilution with DMSO.

### Self‐Assembly of ANFs from PPTA Polyanions

The PPTA polyanion solution was diluted to 0.1 mg mL^−1^ with DMSO and then mixed with water or methanol at a volume ratio of 30:1 and 3:1, respectively.

### Wet Spinning

PPTA polyanion solutions with concentrations of 5, 8, and 10 mg mL^−1^ were used in the wet spinning process using a 5 mL syringe with a needle. The spinning speeds were controlled at 10 and 20 mL h^−1^. After immersion in water as the coagulation solution for 48 h, the resulting hydrogel fibers were either post‐stretched by a roller at 20 r min^−1^ to reach a pre‐determined stretching ratio or dried directly at room temperature with both ends fixed in air. For the synthesis of composite fibers, the wet spinning conditions were the same, except that the coagulation solution was changed from water to Fe^3+^ solutions (0.005 and 0.01 mol L^−1^). The immersion was carried out at 30 °C for durations of 4, 6, and 12 h.

### Characterization

The morphologies and microstructures of the fibers were examined using a scanning electron microscope (SEM, Hitachi SU8020, Japan) at an acceleration voltage of 3 kV with a working distance of 6–8 mm. For the brittle fracture of fiber sections, the fibers were immersed in liquid nitrogen for 30 min and then broken using tweezers below the liquid level. All samples were mounted on an aluminum platform with carbon and copper tapes. Prior to SEM analysis, the surface was sputtered with a Pt layer to improve conductivity. A transmission electron microscope (TEM, Talos F200S G2) at a 200 kV acceleration voltage was used for further structural characterization. For TEM characterization of ANF self‐assembly, aliquots of the DMSO solutions (both with and without water addition) were deposited directly onto TEM grids. For TEM characterization of composite fibers, to avoid damage to the internal structure, a section of the hydrogel fiber was dispersed in water using low‐frequency ultrasound treatment. The solution was then dropped onto a copper mesh with carbon film using a 20 µL pipet. Magnetic hysteresis curves were collected using a superconducting quantum interferometer (SQUID, MPMS XL5) from Lake Shore Cryotronics, Inc., with a maximum applied magnetic field of ±2 T at 298 K. Fourier transform infrared spectroscopy (FTIR, Bruker VERTEX 80v) was performed with a scanning speed of 2 mm s^−1^ and a spectral range of 400–4000 cm^−1^. To analyze spectral changes associated with fracture, all fragmented specimens from multiple tensile tests were collected and characterized. X‐ray photoelectron spectroscopy (XPS) was conducted using an ESCALAB 250Xi electron energy spectrometer (Thermo Fisher Scientific), with Al Kα (1486.6 eV) as the X‐ray excitation source. Thermogravimetric analyses (TGA) were conducted with a TA Instrument (Netzsch STA 449 F5, Germany) in the temperature range from 35 to 800 °C under a nitrogen atmosphere at a heating rate of 10 °C min^−1^. Mechanical properties were assessed using a universal tensile stretching apparatus (WDW‐1T, China) equipped with a 100 N load cell (Transcell BAB‐10MT). Single‐fiber tensile behavior was measured at a strain rate of 1 mm min^−1^ and a gauge length of 10 mm, with a pretension of 0.01 N. The fiber diameters were measured using an optical microscope (HVA‐1000A, China). Nanoindentation tests were performed with a nanoindenter (iMicro, KLA‐Nanosurf) using a Berkovich diamond indenter at 25 °C and 40% relative humidity. The tip was calibrated with molten silica, and thermal drift was kept below 0.05 nm s^−1^. The modulus and hardness were obtained under continuous stiffness measurement mode with a penetration depth of 2000 nm. Samples for nanoindentation tests were prepared by embedding vertically aligned fibers in epoxy resin, which were treated with a polishing machine (UNIPOL‐802) to expose the cross‐sections. Wide‐angle X‐ray diffraction (XRD, Bruker D8 Advance) and wide‐ and small‐angle X‐ray scattering (WAXS/SAXS, Xeuss3.0, France) were used to study the orientation, crystallinity, and crystal size. XRD and SAXS measurements were conducted with a Cu Kα radiation source (wavelength of 1.5418 Å). The sample was positioned 100 mm from the detector for WAXS and 900 mm for SAXS. Both WAXS and SAXS measurements were collected for 300 s. The in situ SAXS test was performed on a Linkam MFS350 test bed at 300 K with a detector distance of 100 mm, a stretching rate of 15 µm s^−1^, and a collection time of 300 s for each 3% increase in strain. During testing, X‐ray beams were used to irradiate a vertically aligned fiber bundle through an aperture with a diameter of 0.5 mm.

To determine the degree of orientation based on the (200) reflection, the 2D WAXS patterns were integrated to generate an azimuthal angle curve using FIT2D software. One of the two peaks in the curve was then fitted using Origin 2021 to calculate Herman's orientation factor (f), according to Equations (1) and (2):

(1) $$f=\frac{3\left(\cos2\phi \right)-1}{2}\;$$

(2) $$\cos2\phi =\frac{\int_{0}^{\pi /2}I(\phi )\mathrm{co}s^{2}\phi \sin\phi d\phi }{\int_{0}^{\pi }I(\phi )d\phi }\;$$

where ϕ represents the azimuthal angle and I(ϕ) denotes the 1D intensity distribution as a function of ϕ. The value of cos2ϕ was calculated by integrating the intensity of the specific 2θ diffraction peak along ϕ using the aforementioned equation.

The crystallinity and crystal size were determined and calculated using MDI Jade 6.0 software. Crystallinity (x_c_) was calculated from the XRD patterns using the Jade 6.0 peak‐fitting procedure, based on the following equation (Equation 3):

(3) $$x_{c}=\frac{A_{\mathrm{crys}}}{A_{\mathrm{crys}}+A_{\mathrm{amor}}}\;$$

where A_crys_ and A_amor_ represent the areas corresponding to the crystalline and amorphous phases, respectively.

The crystal size was calculated from XRD patterns using the Jade 6.0 peak‐fit procedure and the Scherrer equation (Equation 4):

(4) $$L=\frac{k\lambda }{\beta \cos\theta }\;$$

where L is the crystal size, λ is the X‐ray wavelength (1.5418 Å), β is the full width at half‐maximum (FWHM) of the selected peak, θ is the Bragg angle, and k is the Scherrer constant, which was taken as 0.94.

The content of β‐FeOOH (X_β‐FeOOH_) in the composite fibers was calculated according to the following equations (Equations (5), (6), and 7):

(5) $$W_{\mathrm{ANFs}}A+W_{\beta -\mathrm{FeOOH}}B=W_{c}C\;$$

(6) $$W_{\mathrm{ANFs}}+W_{\beta -\mathrm{FeOOH}}=W_{c}\;$$

(7) $$X_{\beta -\mathrm{FeOOH}}=\frac{C-A}{B-A}\;\times 100\%\;$$

where W_ANFs_, W_β‐FeOOH_, and W_c_ are the mass of ANFs, β‐FeOOH, and composite fibers, respectively. A, B, and C are the remaining mass percentages of ANFs, β‐FeOOH nanowhiskers, and composite fibers after the TGA test, respectively.

The birefringence was calculated by using the Sénarmont compensation method. In a typical test, the analyzer was rotated so that the fiber in the field of view became completely black. The angle rotated at this time is the angle θ. Then, the birefringence of the fiber can be calculated as follows (Equation 8):

(8) $$\Delta n=\frac{\theta \lambda }{\pi d}$$

where λ is the wavelength of incident monochromatic light (λ = 589.3 nm), and d is the fiber diameter.

### Calculation of Dipole Moments

The PPTA primitive cells were expanded by 3 units along the c‐axis, while β‐FeOOH primitive cells were expanded by 3 units along the b‐axis. The periodic structures were geometrically optimized by using the CAMPASS force field in the Forcite module in Materials Studio. Then the periodic structure was removed to show the dipole moment.

### Calculation of Cohesive Energy Density (CED)

The crystal structures of Nylon, Nomex, and PPTA were used in the atomic molecular dynamics simulations. The Forcite module in Materials Studio, along with the COMPASS force field, was employed to perform geometric optimization and calculate the energy of the crystal and the single chain, respectively. The CED was calculated using Equation (9):

(9) $$\mathrm{CED}=\frac{\sum_{i=1}^{n}U_{i}-U_{\mathrm{condense}}}{V}$$

where i represents the individual chains that make up the system, U_i_ is the average energy of the isolated chains i, U_condense_ is the average potential energy of the entire simulated system in the condensation stage, and V is the volume of the entire simulation system.

## Conflict of Interest

The authors declare no conflict of interest.

## Supporting information



Supporting Information

## Data Availability

The data that support the findings of this study are available in the supplementary material of this article.
