# Supplementary material for: Limpet‐Inspired Multifunctional Composite Fibers with Exceptional Mechanical Performance From Self‐Assembled Aramid Nanofibers
Source: Adv Sci (Weinh). 2025 Aug 26;12(39):e09321. doi: 10.1002/advs.202509321 (PMC12533336; doi:10.1002/advs.202509321)
Supplement: Supplementary file 1 — Supporting Information [file ADVS-12-e09321-s001.docx]

Supporting Information

**Limpet-Inspired Multifunctional Composite Fibers with Exceptional Mechanical Performance from Self-Assembled Aramid Nanofibers**

*Tiantian Ren, Shouhua Feng, and Ming Yang**

The State Key Laboratory of Inorganic Synthesis and Preparative Chemistry, College of Chemistry, Jilin University, Changchun 130012
E-mail: mingyang@jlu.edu.cn

**Figure S1.** XRD patterns of PPTA powder and Kevlar.

**a**

**b**

**Figure S2.** Statistical analyses of diameters of self-assembled ANFs after the addition of varying amounts of water: a) DMSO/water (v/v) ratio of 30:1, and b) DMSO/water (v/v) ratio of 3:1.


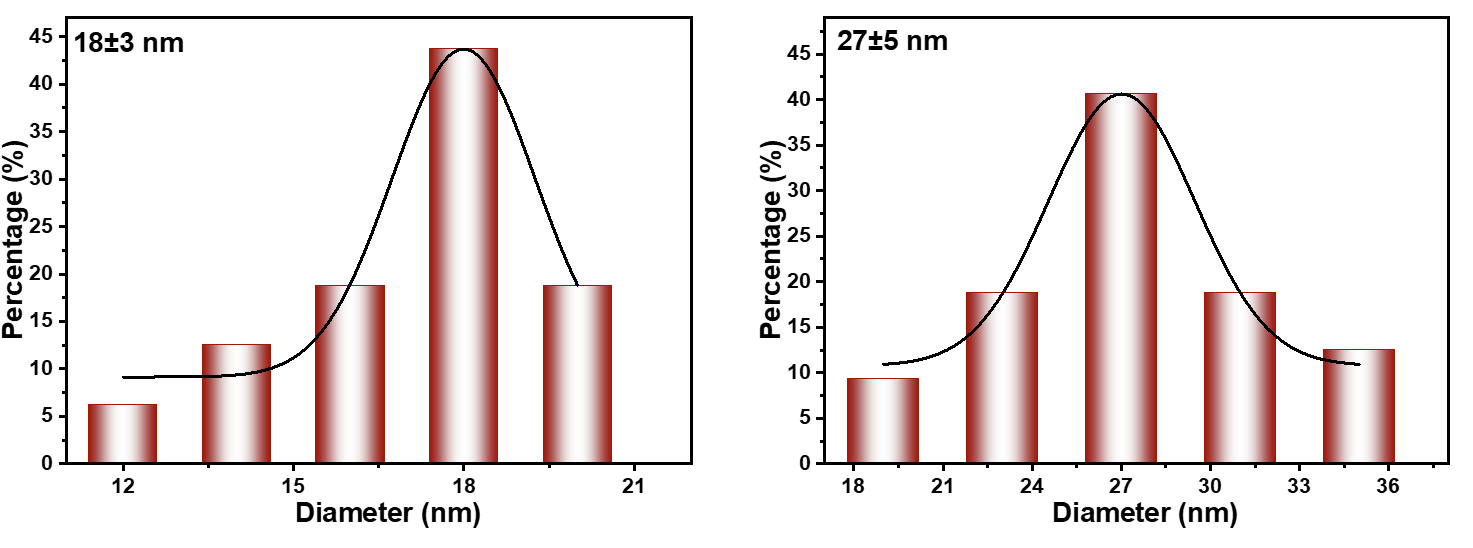


**Figure S3.** Guinier approximation of DMSO solutions of PPTA polyanions with different water additions.

**Figure S6.** Cross-sectional SEM images of hydrogel fibers after a freeze-drying process: a) no post-stretching, and b) with a stretching ratio of 10%.


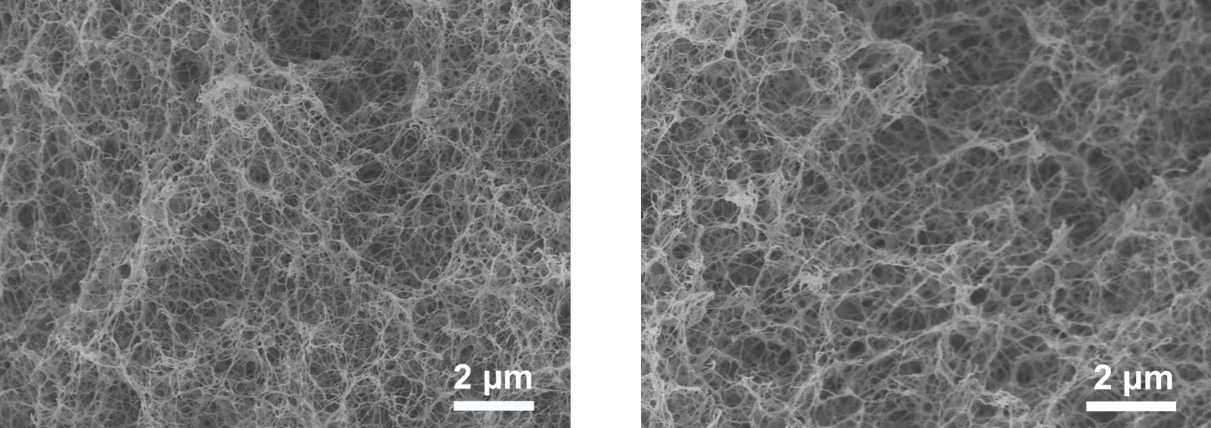


**a**

**b**

**Figure S5.** A TEM image of ANFs obtained using methanol as the nonsolvent.


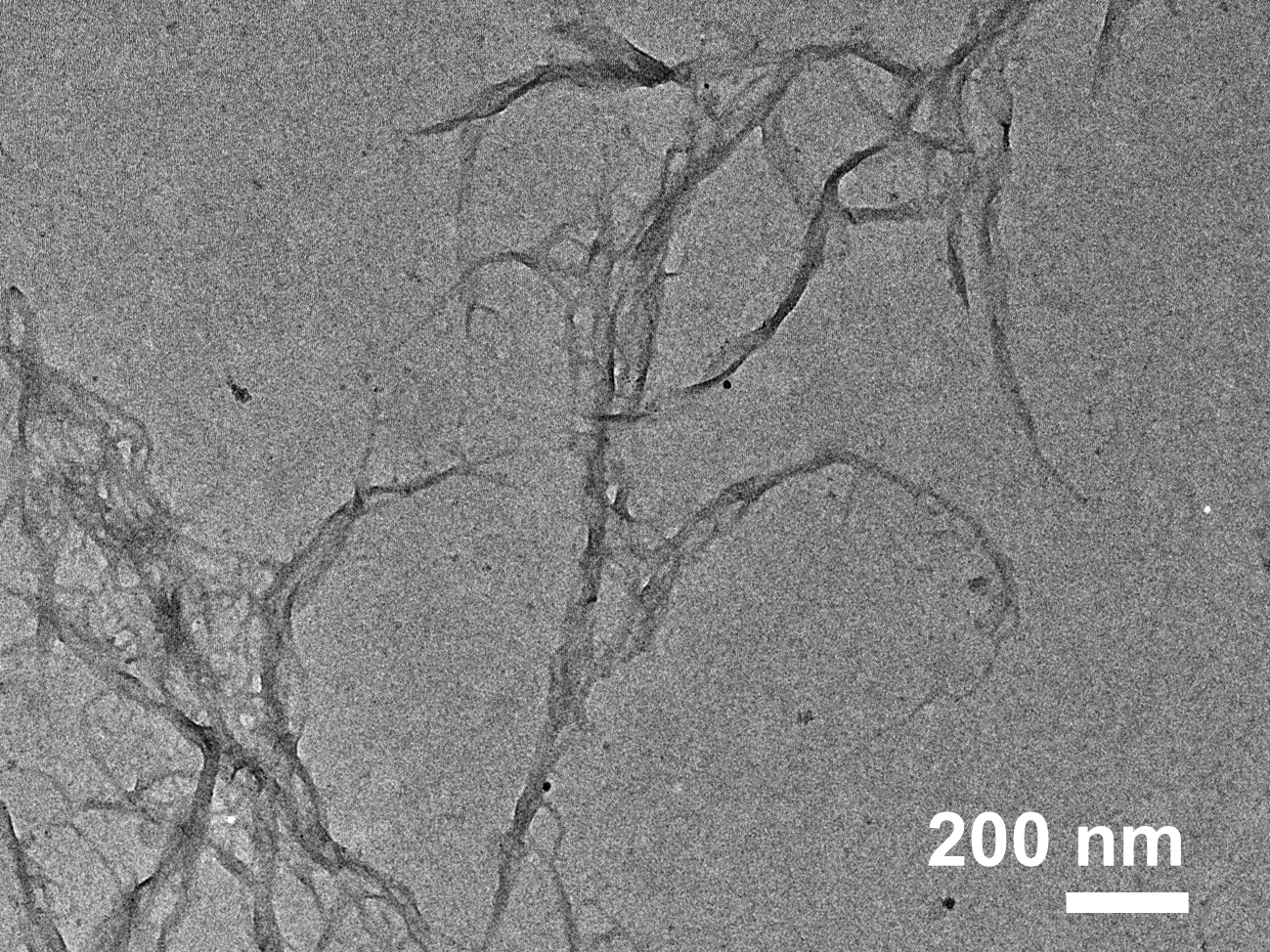

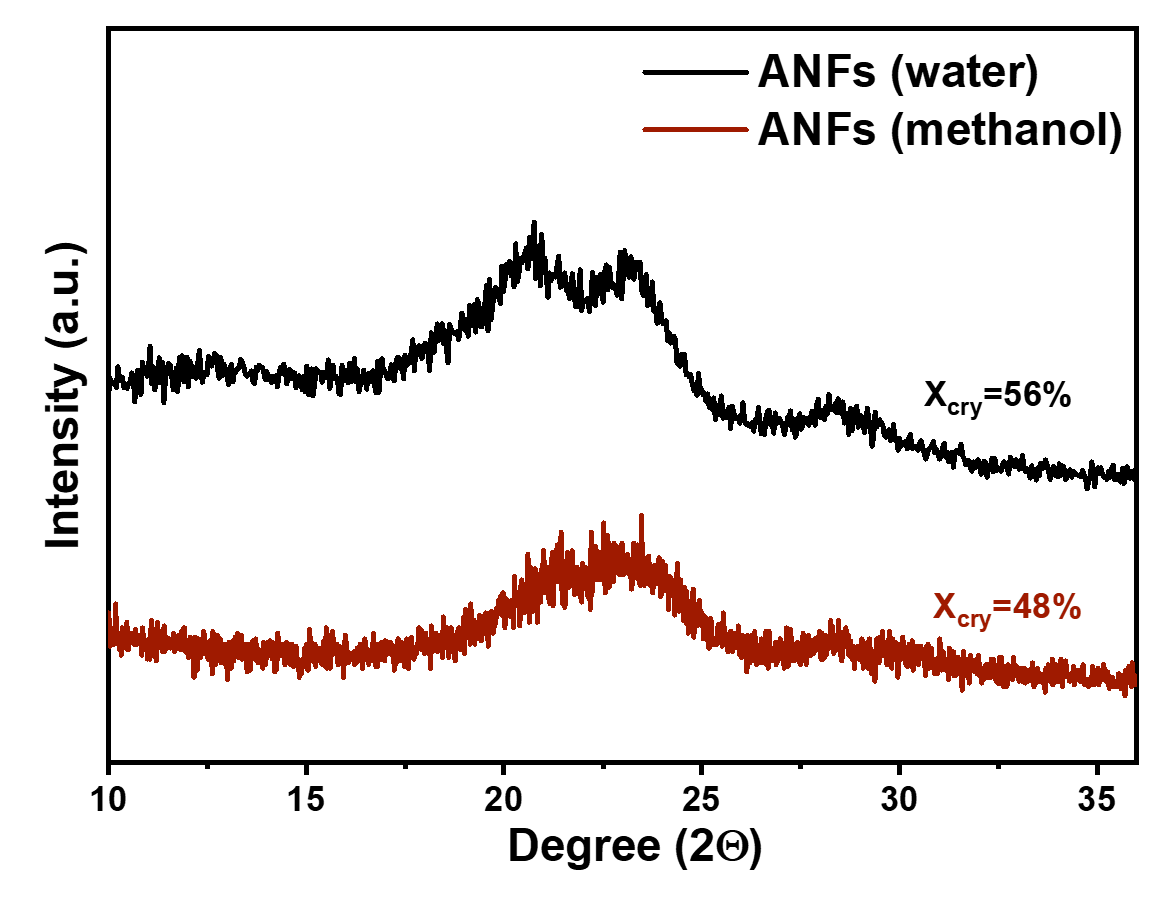


**Figure S4.** XRD patterns of ANFs obtained using excess amounts of water and methanol as the nonsolvent. The calculated crystallinity is 56% and 48%, respectively.

**Table S1. Tensile properties of aramid fibers obtained under different wet spinning conditions.**

**Table S2. Structural parameters for different aramid fibers.**

**Figure S7. 2D WAXS patterns of aramid fibers.** The dope concentration and the spinning speed are a) 5 mg/mL and 10 mL/h, b) 8 mg/mL and 10 mL/h, and c) 10 mg/mL and 10 mL/h, respectively. The spinning condition for d) is the same as a), except that a post-stretching process was applied with a stretch ratio of 10%.


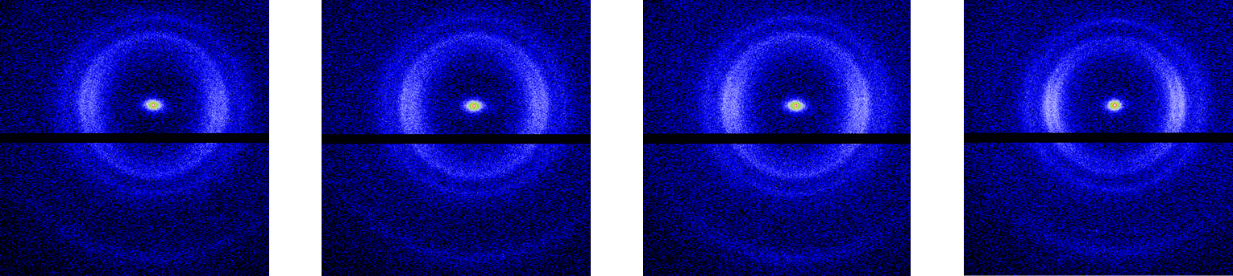


**a**

**b**

**c**

**d**

**Figure S8.** Stress-strain curves of aramid fibers obtained using PPTA powder and Kevlar as the precursor, respectively. The dope concentration is 5 mg/mL, the spinning speed is 10 mL/h, and the post stretching ratio is 10%.

**Figure S10.** XRD patterns of composite fibers obtained after different immersion time in the Fe^3+^ solution.


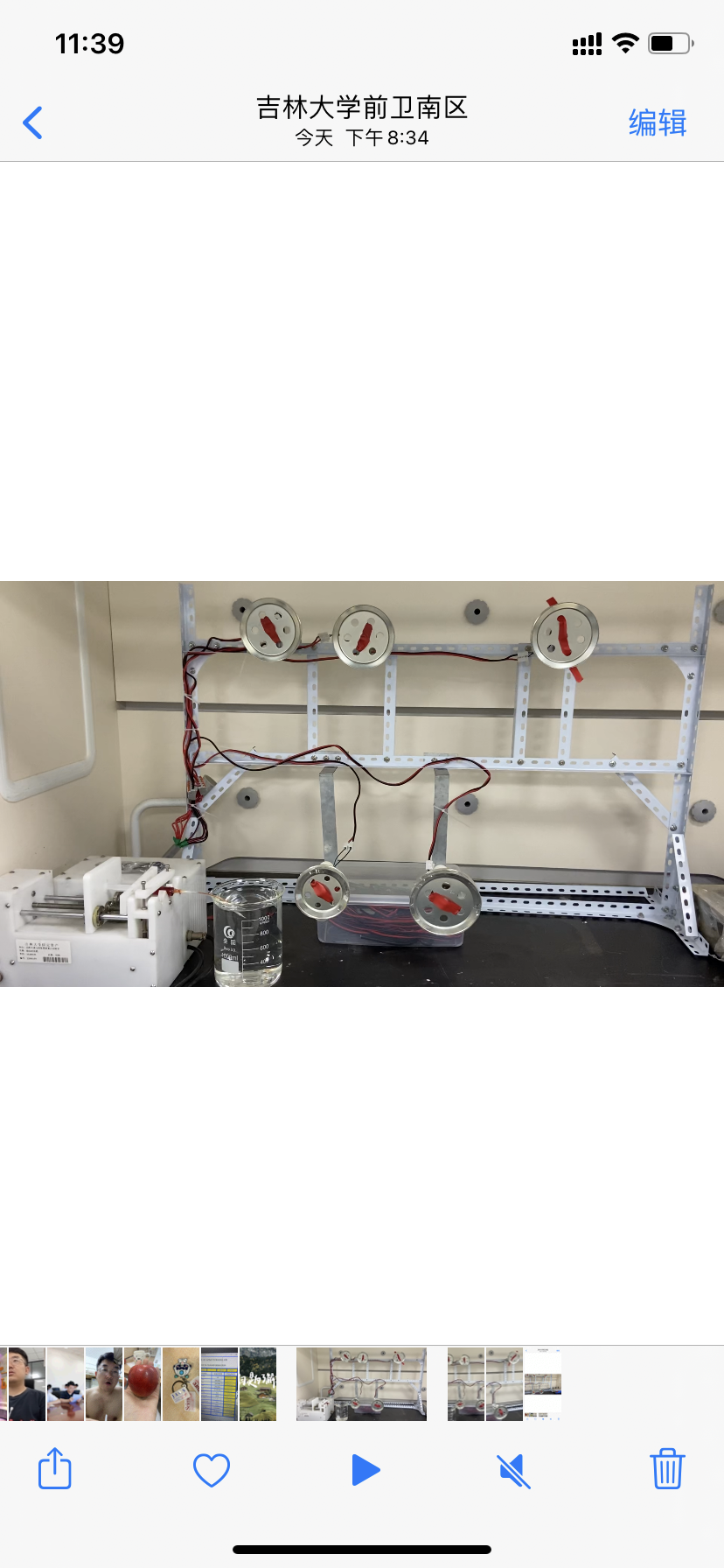


**Figure S9.** A home-built device for the continuous spinning.

**Table S3. Comparisons of mechanical properties with literature results.**


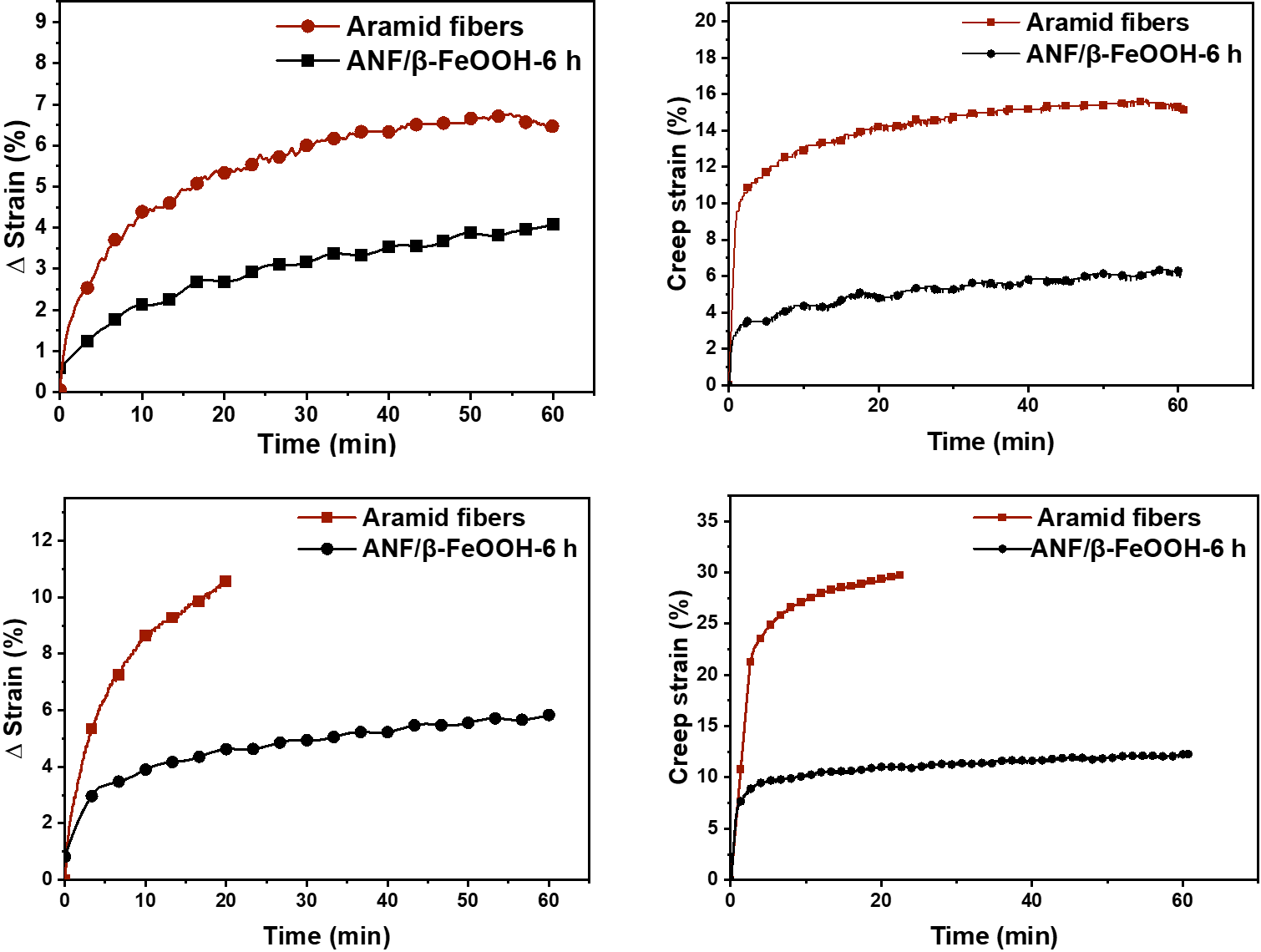


**a**

**b**

**Figure S11.** Time-dependent △strain and creep strain for aramid fibers and ANF/β-FeOOH-6 h when the constant force is a) 30% and b) 60% of the failure load of aramid fibers.

**Figure S12.** UV-vis spectra of β-FeOOH nanowhiskers.


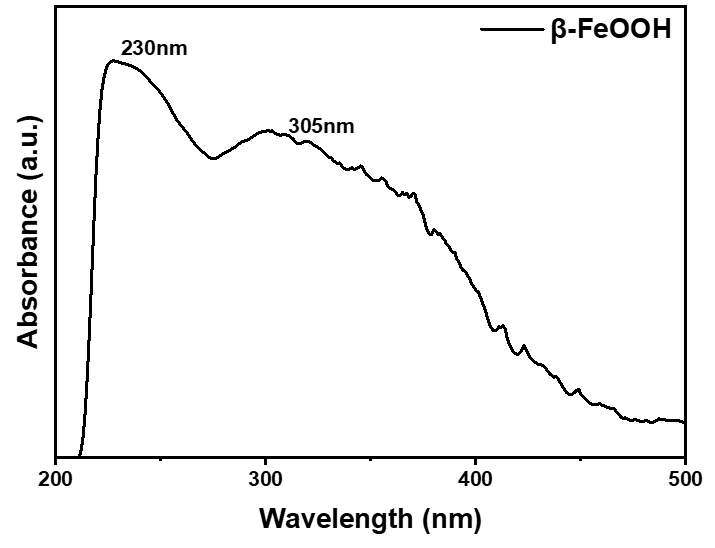

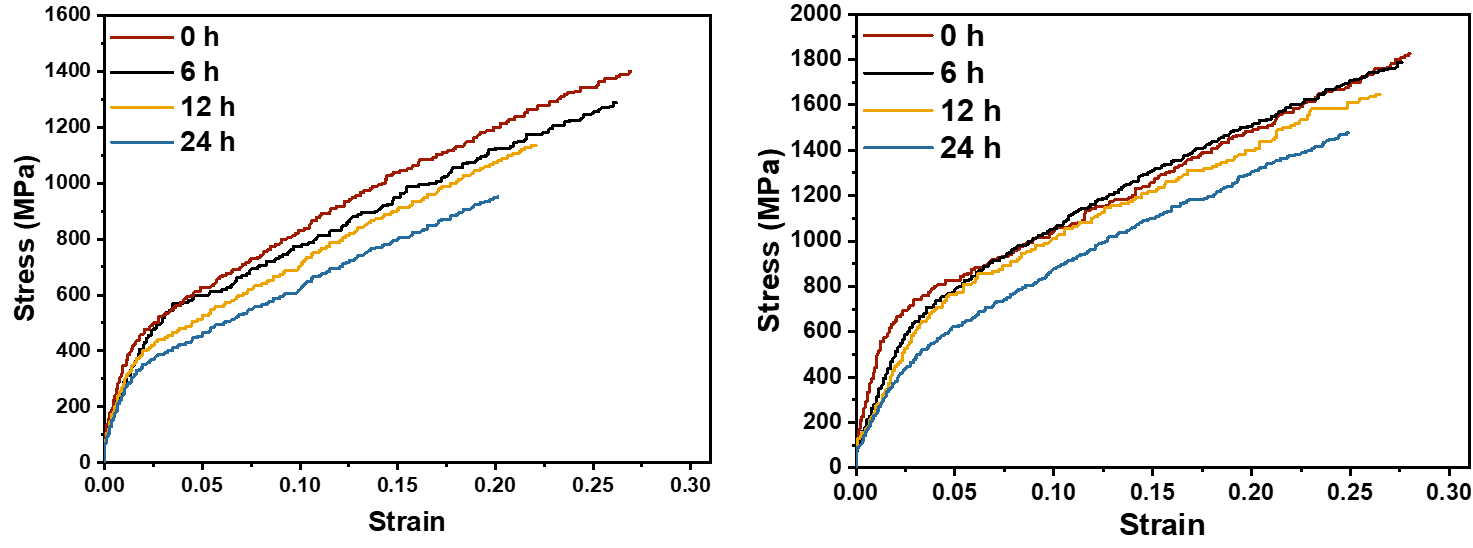


**a**

**b**

**Figure S13.** Stress-strain curves of a) aramid fibers and b) ANF/β-FeOOH-6 h after ultraviolet radiation for different lengths of time.


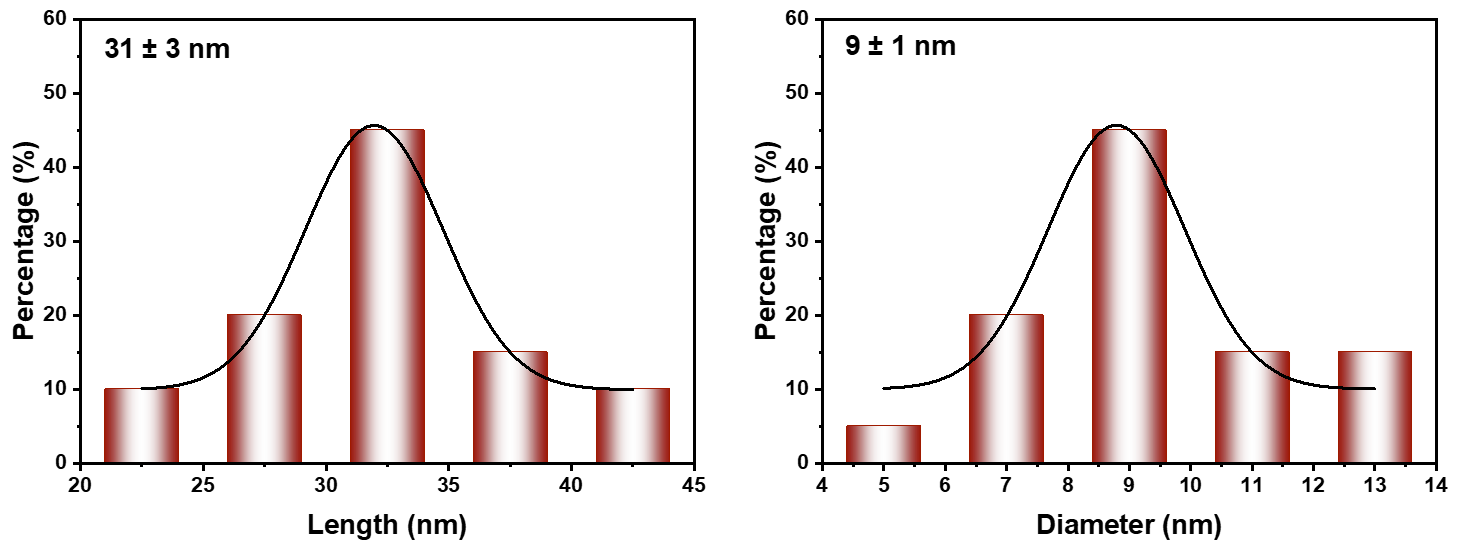


**a**

**b**

**Figure S14.** Statistical analyses of a) the length and b) the diameter of β-FeOOH nanowhiskers in ANF/β-FeOOH-6 h.

**Table S4. Tensile properties of aramid fibers and** **ANF/β-FeOOH-6 h after ultraviolet radiation.**


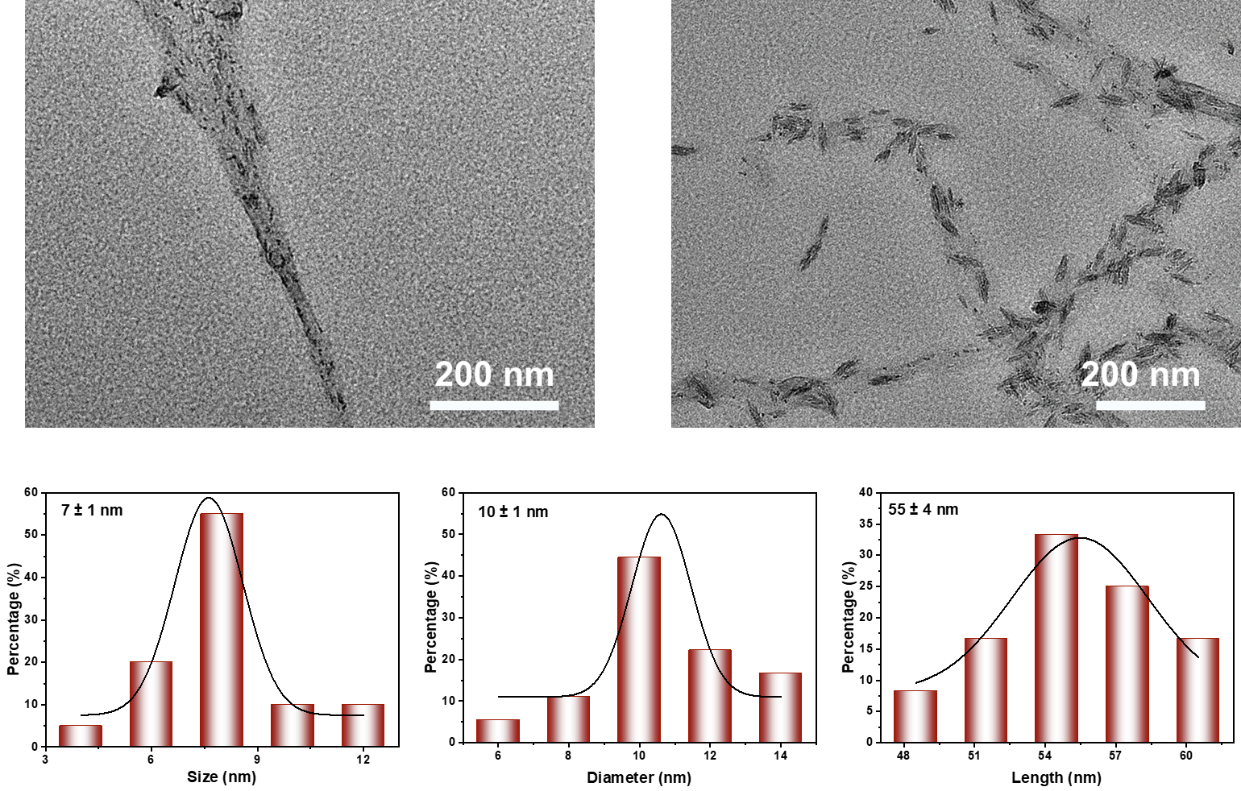


**Figure S15.** TEM images of hydrogel fibers after a) 4-h and b) 12-h immersion in the Fe^3+^ solutions. Statistical analyses of c) nanoparticle size after 4-h immersion, d) the diameter and e) the length of β-FeOOH nanowhiskers after 12-h immersion.

**a**

**b**

**c**

**d**

**e**

**Figure S16.** TGA curves of different fibers.

**Table S5. Tensile properties of composite fibers obtained using the 0.01 mol/L Fe^3+^ solution as the coagulation bath. Post-stretching ratio of 10% was applied.**


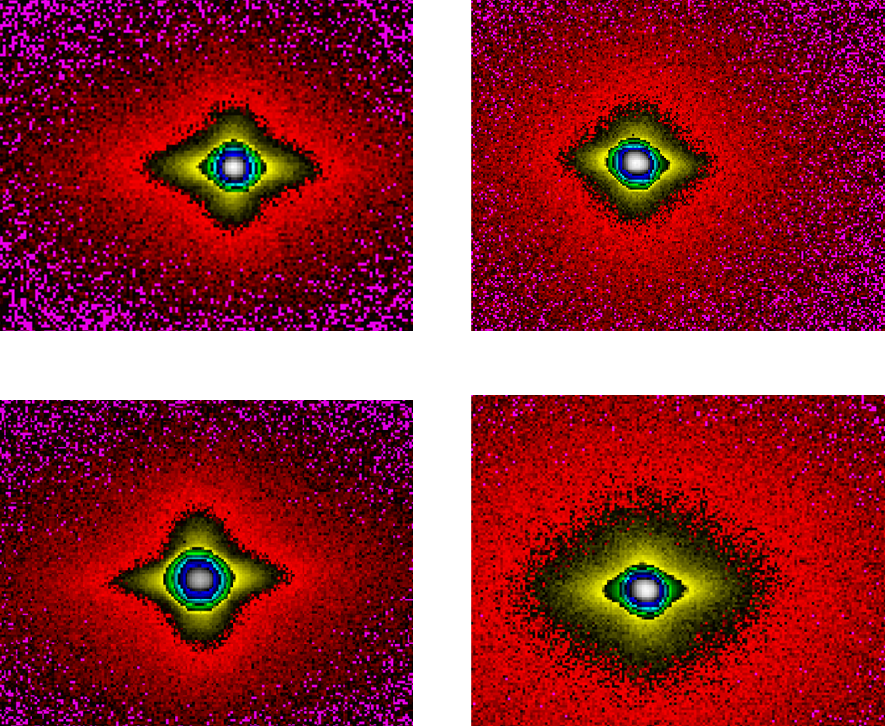


**a**

**d**

**c**

**Figure S17.** 2D SAXS patterns of a) aramid fibers, b) ANF/β-FeOOH-4 h, c) ANF/β-FeOOH-6 h and d) ANF/β-FeOOH-12 h.

**b**


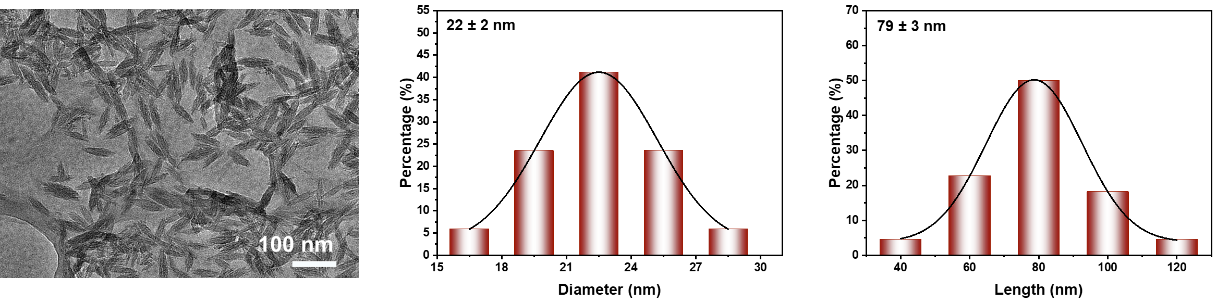


**a**

**Figure S18.** a) A TEM image of hydrogel fibers obtained after immersion in the Fe^3+^ solution (0.01mol/L) for 12 h. Statistical analyses of b) the diameter and c) the length of β-FeOOH nanowhiskers.

**b**

**c**

**Figure S20.** IR spectra of aramid fibers and ANF/β-FeOOH-6 h.

**Figure S19.** XRD patterns of aramid fibers.

**Figure S21.** High resolution O1s spectrum of aramid fibers and ANF/β-FeOOH-6 h.


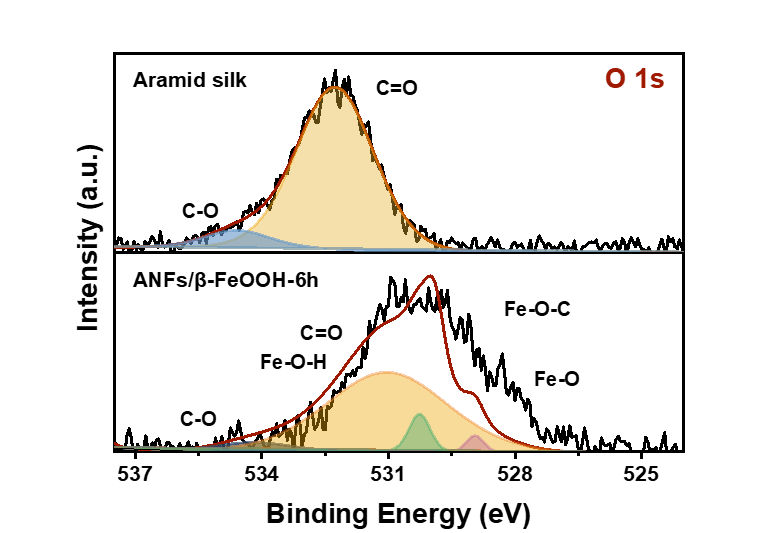


**Figure S22.** XRD patterns of ANFs obtained using excess amounts of water and Fe^3+^ solutions (0.005 M).The calculated crystallinity is 56% and 43%, respectively.

**Figure S23.** A cross-sectional SEM image of aramid fibers after stretching-induced failure.


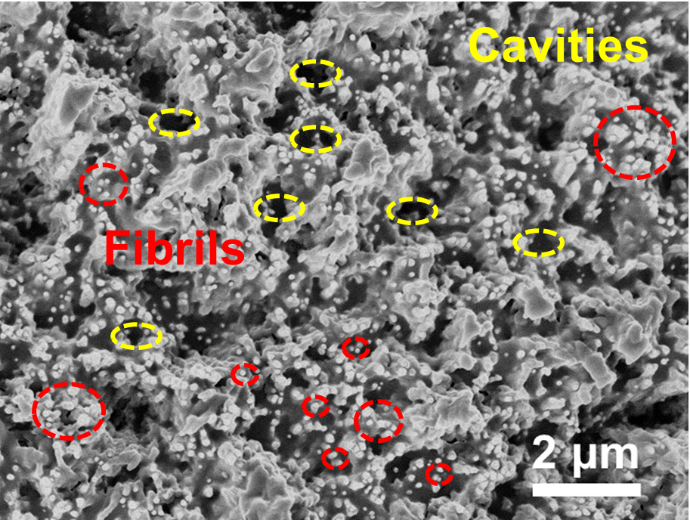


**Figure S24.** IR spectra of aramid fibers before and after stretching-induced failure.


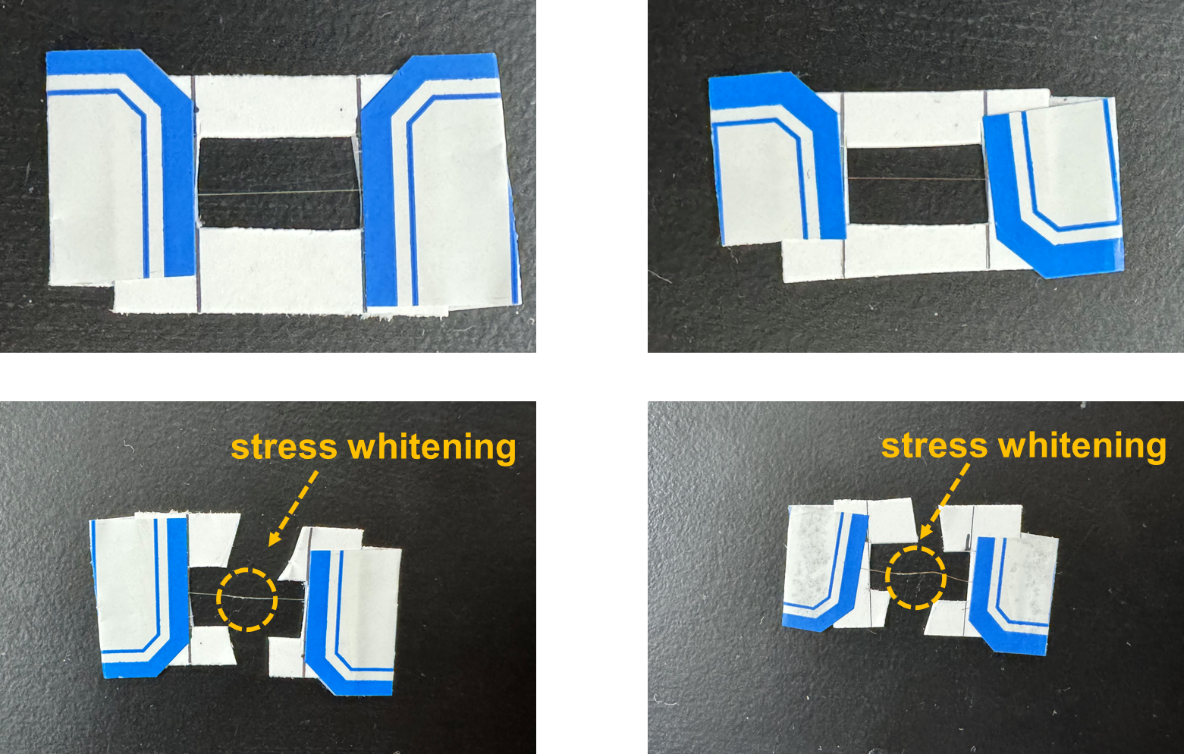


**b**

**Figure S25.** Optical images of a) aramid fibers and b) ANF/β-FeOOH-6 h before stretching. Optical images of c) aramid fibers after stretching to a strain of 18% and d) ANF/β-FeOOH-6 h after stretching to a strain of 21%.

**a**

**c**

**d**

**Table S6. Structural parameters for aramid fibers and ANF/β-FeOOH-6 h under different strains.**

**Figure S28.** Representative 1D WAXD intensity profiles of aramid fibers under different strains.

**Figure S27.** Representative 2D-WAXD plots of ANF/β-FeOOH-6 h under different strains at a stretching rate of 1mm/min.


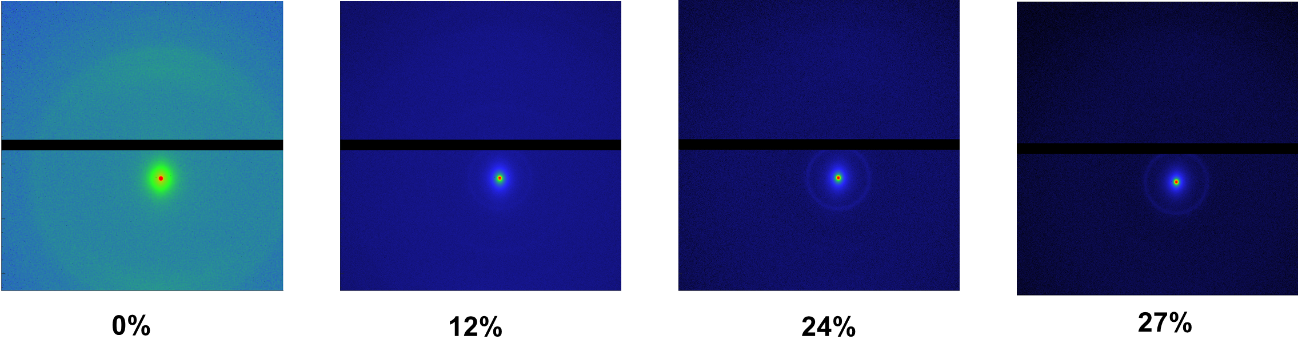

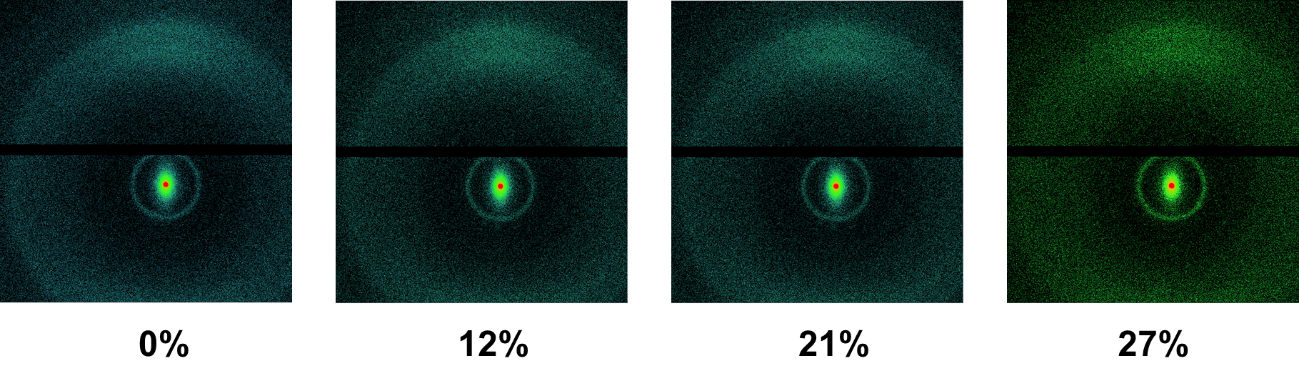


**Figure S26.** Representative 2D-WAXD plots of aramid fibers under different strains at a stretching rate of 1mm/min.

**Figure S29.** Representative 1D WAXD intensity profiles of ANF/β-FeOOH-6 h under different strains.


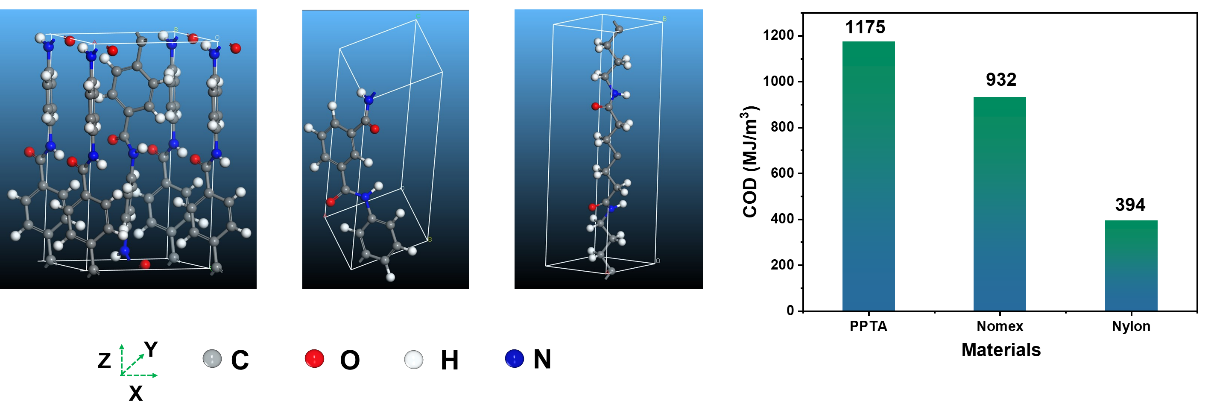


**a**

**b**

**c**

**Figure S30.** Different crystal models of a) PPTA, b) Nomex and c) Nylon. d) Calculated cohesion energy density (CED) of PPTA, Nomex, and nylon.

**d**
